# The role of insulators and transcription in 3D chromatin organization of flies

**DOI:** 10.1101/gr.275809.121

**Published:** 2022-04

**Authors:** Keerthi T. Chathoth, Liudmila A. Mikheeva, Gilles Crevel, Jareth C. Wolfe, Ioni Hunter, Saskia Beckett-Doyle, Sue Cotterill, Hongsheng Dai, Andrew Harrison, Nicolae Radu Zabet

**Affiliations:** 1 School of Life Sciences, University of Essex, Colchester CO4 3SQ, United Kingdom;; 2 Blizard Institute, Barts and The London School of Medicine and Dentistry, Queen Mary University of London, London E1 2AT, United Kingdom;; 3 Department of Mathematical Sciences, University of Essex, Colchester CO4 3SQ, United Kingdom;; 4 Department Basic Medical Sciences, St. Georges University London, London SW17 0RE, United Kingdom;; 5 School of Computer Science and Electronic Engineering, University of Essex, Colchester CO4 3SQ, United Kingdom

## Abstract

The DNA in many organisms, including humans, is shown to be organized in topologically associating domains (TADs). In *Drosophila*, several architectural proteins are enriched at TAD borders, but it is still unclear whether these proteins play a functional role in the formation and maintenance of TADs. Here, we show that depletion of BEAF-32, Cp190, Chro, and Dref leads to changes in TAD organization and chromatin loops. Their depletion predominantly affects TAD borders located in regions moderately enriched in repressive modifications and depleted in active ones, whereas TAD borders located in euchromatin are resilient to these knockdowns. Furthermore, transcriptomic data has revealed hundreds of genes displaying differential expression in these knockdowns and showed that the majority of differentially expressed genes are located within reorganized TADs. Our work identifies a novel and functional role for architectural proteins at TAD borders in *Drosophila* and a link between TAD reorganization and subsequent changes in gene expression.

Topologically associating domains (TADs) provide a fundamental unit for chromosome organization (Dixon et al. 2012; Sexton et al. 2012) and are widely conserved across species (Vietri Rudan et al. 2015) as well as during different developmental stages (Ghavi-Helm et al. 2014; Dixon et al. 2015), suggesting that they have a functional role. Furthermore, in *Drosophila* cells, changes in the 3D organization of DNA after heat stress have been found to correlate with transcriptional changes (Li et al. 2015). Recent evidence points to defective 3D architecture as a major contributor for diseases, developmental defects, and even aging (Chandra et al. 2015; Lupiáñez et al. 2015; Flavahan et al. 2016; Hnisz et al. 2016; Sun et al. 2018; Kraft et al. 2019; Akdemir et al. 2020). These results suggest that 3D organization of the DNA is important in gene regulation.

There has been significant progress in generating empirical data on chromatin organization in different organisms and tissues, but, despite this, the mechanisms that drive the formation of TAD borders remain unclear. Previous research has shown that TAD borders are enriched in housekeeping genes (Li et al. 2015), developmental enhancers (Cubeñs-Potts et al. 2017), and boundaries of highly conserved genomic regulatory blocks (Harmston et al. 2017). In addition, architectural proteins and insulators are enriched at TAD borders (Van Bortle et al. 2014; Stadler et al. 2017). Two different mechanisms were proposed to be responsible for TAD formation: (1) compartment domains, which are formed by interactions among sequences that contain active or inactive histone modifications; and (2) loop domains that are flanked by CTCF binding sites and are formed by a cohesion-driven loop extrusion mechanism (Rao et al. 2014; Mirny et al. 2019; de Wit 2020). The latter displays a strong loop localized at the top of the TAD, whereas the former lacks this chromatin loop. In mammalian systems, CTCF and cohesin are the main architectural components that are located at TAD borders, and their depletion has been shown to disrupt TADs (Zuin et al. 2014; Nora et al. 2017; Schwarzer et al. 2017). In contrast, in *Drosophila*, several insulator proteins occupy TAD borders, such as CTCF, BEAF-32, Chro, and Cp190 (Van Bortle et al. 2014; El-Sharnouby et al. 2017; Ramírez et al. 2018; Chathoth and Zabet 2019; Matthews and White 2019), but the majority of TADs lack the chromatin loop at the top of the TAD, suggesting a prevalence of the compartment domains (Matthews and White 2019; Rowley et al. 2019). In particular, previous research has identified strong enrichment of BEAF-32 at TAD borders in *Drosophila* (Van Bortle et al. 2014; Ramírez et al. 2018; Wang et al. 2018; Chathoth and Zabet 2019), but this was more pronounced in cell lines derived from the embryo (Kc167 derived from dorsal closure stage and S2 derived from late embryonic stage) or whole embryos. Nevertheless, there are negligible changes in 3D chromatin organization following *BEAF-32* RNAi knockdown in Kc167 cells (Ramírez et al. 2018) despite the strong enrichment of BEAF-32 at TAD borders. Kc167 cells display saturating levels of BEAF-32 at TAD borders, suggesting that, upon RNAi knockdown, there is potentially still sufficient protein present in the cell to maintain TAD borders (Martin and Zabet 2020). Furthermore, BEAF-32 displays a similar binding motif as another architectural protein in *Drosophila* called Dref (Hirose et al. 1996; Mathelier et al. 2014). When BEAF-32 is depleted, one possibility is that Dref replaces it at TAD borders, and this could explain the lack of changes in 3D organization observed in Kc167 cells.

Two additional proteins, Cp190 and Chro, are enriched at TAD borders (Cubeñs-Potts et al. 2017; El-Sharnouby et al. 2017; Wang et al. 2018). These proteins cannot bind independently to DNA but are recruited mainly by BEAF-32 (Vogelmann et al. 2014), with up to 91% of TAD borders in a *Drosophila* cell line (S2) displaying the presence of BEAF-32 together with either Cp190 or Chro (Wang et al. 2018). Like BEAF-32, the role of Cp190 and Chro at TAD borders is currently unclear.

Recently, the role of TADs in gene regulation has been challenged (Ghavi-Helm et al. 2019; Williamson et al. 2019; Ing-Simmons et al. 2021). In one example, it was shown that changes in TAD borders and changes in transcription are not coupled when investigating a *Drosophila* balancer chromosome containing chromosome rearrangements (Ghavi-Helm et al. 2019). However, the balancer chromosomes display a very small number of rearrangements that result in changes at only a few TAD borders. It is less likely that effects on gene expression will be observed when sampling only a few rearrangements, and one possibility is that more and stronger changes in TADs (e.g., more TAD borders are lost) would allow the observation of changes in gene expression that correlate with reorganizations of TADs.

Here, we depleted BEAF-32 in BG3 cells (derived from the larval central nervous system) using RNAi knockdown and measured the changes in 3D chromatin organization at sub-kilobase resolution together with changes in transcription to dissect the mechanism at TAD borders and evaluate the functional role of TADs. In BG3 cells, BEAF-32 has reduced levels at TAD borders (Chathoth and Zabet 2019), which raises the question of whether a strong depletion combined with the low levels of BEAF-32 is sufficient to affect the borders of the TADs. We also performed double knockdowns of *Cp190/Chro* and *BEAF-32/Dref* using RNAi to disentangle the interactions between different architectural proteins at TAD borders.

## Results

### BEAF-32, Cp190, and Chro have functional roles at TAD borders in BG3 cells

We performed single knockdown of *BEAF-32* and combinatorial knockdown of *Cp190* and *Chro* in BG3 cells followed by in situ Hi-C (Supplemental Fig. S1; Supplemental Tables S1, S2). The knockdowns lead to specific and strong reduction in both the mRNA levels and protein levels and do not affect the cell cycle (the efficiency of knockdown achieved here is similar to the ones reported by other studies in *Drosophila* cells [Supplemental Fig. S1; Schwartz et al. 2012; Ramírez et al. 2018; Zenk et al. 2021]). High-resolution contact maps were generated for both knockdowns. The biological replicates displayed high similarities and were merged for the downstream analysis (Supplemental Fig. S2A). BG3 *BEAF-32*^RNAi^ resulted in loss of long-range interactions and showed an increase in short-range interactions (Supplemental Fig. S2A–C). Likewise, BG3 *Cp190*^RNAi^ *Chro*^RNAi^ also exhibited reduced long-range interactions and increased short-range interactions, but the loss of long-range interactions was less pronounced compared to BG3 *BEAF-32*^RNAi^ (Supplemental Fig. S2A–C).

**Figure 1.**
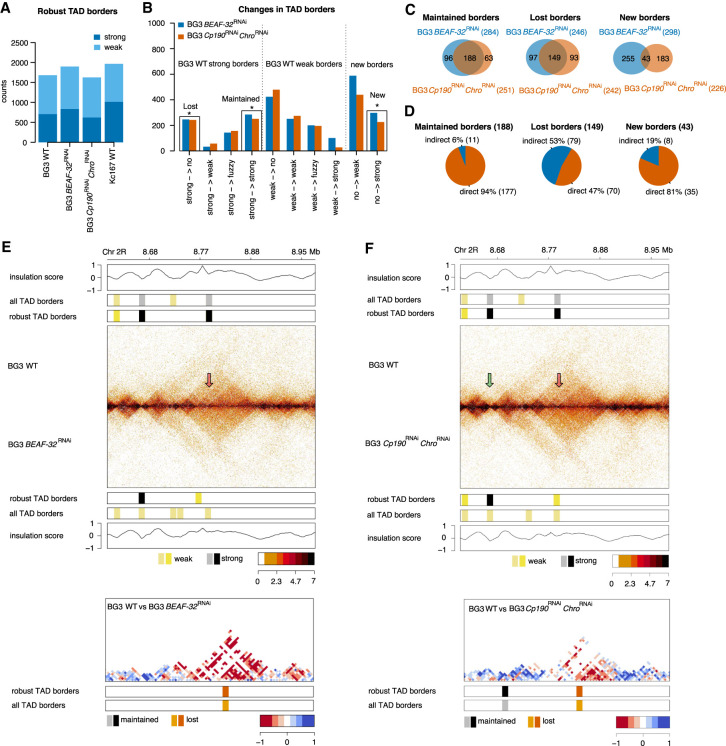
Functional roles of BEAF-32, Cp190, and Chro in TAD organization of BG3 cells. (*A*) Number of robust TAD borders in BG3 cells (WT, *BEAF-32* knockdown, and *Cp190 Chro* double knockdown) (see Supplemental Fig. S3A). We also included the number of TAD borders in Kc167 cells. We split each class of TAD border into two subgroups: strong borders and weak borders, depending on whether the TAD borders can still be detected when increasing the stringency of the TAD-calling algorithm. (*B*) Classification of TAD borders as described in the main text: lost (borders that are strong in WT and completely disappear in the knockdown); maintained (borders that are strong in WT and are maintained strong in the knockdown); and new (borders that appear strong in the knockdown). (*C*) Overlap of lost, maintained, and new borders in the two knockdowns. (*D*) Number and percentage of maintained, lost, and new borders that have direct binding of BEAF-32, Cp190, and/or Chro (see Supplemental Fig. S4A). We considered common borders between the two knockdowns (*BEAF-32* single knockdown and Cp190 and Chro double knockdown). (*E*,*F*) Examples of a genomic region at DpnII restriction size resolution for: (*E*) *BEAF-32*; and (*F*) *Cp190* and/or *Chro* knockdowns. Darker colors indicate more contacts retrieved by in situ Hi-C. Green arrow indicates maintained borders and red arrows lost borders. From *top* to *bottom*, we plot the insulation score, TAD borders in the full data set (gray are strong and yellow are weak), TAD borders recovered both in the full and down-sampled data set (black are strong and yellow are weak), and contact map in WT cells. We also plot a mirror plot in the knockdowns (*BEAF-32* knockdown or *Cp190 Chro* double knockdown) and log_2_fold change between WT and knockdown. To compute the log_2_FC, we followed the steps and parameters recommended in the diffHiC package (Lun and Smyth 2015). Briefly, we considered individual replicates and used the edgeR package (Robinson et al. 2010) to compute the log_2_FC in 5-kb bins.

Several papers have proposed that BEAF-32, Cp190, and Chro control the borders of TADs (Van Bortle et al. 2014; Ramírez et al. 2018; Wang et al. 2018). Here, we used HiCExplorer (Ramírez et al. 2018) and identified between 2000 and 2600 TADs at DpnII resolution (∼529 bp) (see Supplemental Table S2; Methods), which is consistent with other studies (Cubeñs-Potts et al. 2017; Ramírez et al. 2018; Chathoth and Zabet 2019). TAD borders were classified into weak and strong borders depending on whether they can be detected with increasing stringency of the TAD-calling algorithm, with strong borders being detected even with the more stringent parameters (see Fig. 1A; Methods). To investigate the robustness of these TAD borders to differences in the size of Hi-C libraries, we down-sampled all Hi-C libraries by 20% and repeated the analysis (see Supplemental Fig. S3A; Chathoth and Zabet 2019). Of the 989 strong TAD borders in WT cells, 706 are robust, meaning they are recovered in both full and down-sampled data sets (Supplemental Fig. S3A).

Compared to WT BG3 cells, out of all strong borders (706), 188 borders were maintained and 149 were lost in both knockdowns (BG3 *BEAF-32*^RNAi^ and BG3 *Cp190*^RNAi^ *Chro*^RNAi^), with the rest of the strong borders in WT cells either not displaying the same trend in both knockdowns, moving only within 2 kb (fuzzy) or only moderately weakening (see Fig. 1B,C; Methods). Supplemental Figure S3B and C confirms that difference in insulation between maintained and lost borders increases in both knockdowns.

In both knockdowns. approximately 150 strong borders and 200 weak borders changed their position within 2 kb and we called them fuzzy borders. Twenty-five percent of the fuzzy strong borders are common between the two knockdowns (BG3 *BEAF-32*^RNAi^ and BG3 *Cp190*^RNAi^ *Chro*^RNAi^), which is significantly lower compared to maintained borders (66%; Fisher's exact test *P*-value = 8.7 × 10^−6^). Nevertheless, these common fuzzy borders display a similar number of BEAF-32 binding sites as maintained borders, which is higher than at lost borders (Supplemental Fig. S4I). Furthermore, the majority of common fuzzy borders display binding of BEAF-32, Cp190, and/or Chro and thus are direct borders (Supplemental Fig. S4J). In addition, approximately one-quarter of 975 weak borders from BG3 WT cells were maintained as weak borders in the two knockdowns, but only a negligible number of borders converted from strong to weak or vice versa (Fig. 1B).

Next, in order to distinguish between direct and indirect effects, we evaluated how many of the maintained and lost robust borders have BEAF-32, Chro, or Cp190 ChIP peaks (Riddle et al. 2011; Schwartz et al. 2012) in their vicinity in WT cells. Figure 1D shows that the majority of maintained TAD borders (94%) are direct targets of the three proteins, but only half of the lost TAD borders (47%) are direct targets (also see Supplemental Fig. S4A). To further confirm that the direct maintained and direct lost TAD borders are indeed controlled by the three architectural proteins, we analyzed ChIP data in two RNAi knockdowns in BG3 cells (*BEAF-32* and *Cp190* single knockdowns) (Schwartz et al. 2012) and found that the majority of maintained TAD borders (70%) retain BEAF-32 or Cp190 upon knockdown, whereas most of the lost borders (70%) lose binding of these architectural proteins after knockdown (Supplemental Fig. S4B,C).

Some regions displayed high conservation of the TAD structure organization, whereas others showed reorganization (Fig. 1E,F). We observed that a loss of a TAD border could result in either movement of the TAD borders or aggregation of two TADs (Fig. 1F). We also found new border formation in both knockdowns, ranging from 400 to 600 weak borders and 200 to 300 strong borders. The majority of these new borders moved more than 2 kb in the knockdowns compared to WT (Supplemental Fig. S5). A small proportion of the new TAD borders result in splitting the original TAD in two separate TADs (Supplemental Fig. S5). Out of all the new borders, only 43 were common between both knockdowns (Fig. 1C). This may be explained by the fact that Chro and Cp190 are able to bind chromatin independent of BEAF-32 (Schwartz et al. 2012). Most of these new borders have BEAF-32, Chro, or Cp190 ChIP peaks in their vicinity and retain BEAF-32 or Cp190 upon knockdown (Fig. 1D; Supplemental Fig. S4A–C). Note that whereas the RNAi is efficient, it does not lead to complete removal of the architectural proteins (see Supplemental Fig. S1D). To identify the roles of BEAF-32, Cp190, and Chro at TAD borders, we focused on two groups: (1) maintained borders (robust TAD borders that are strong in WT cells and are maintained strong in both knockdowns); and (2) lost borders (robust TAD borders that are strong in WT cells and are lost in the two knockdowns).

Lost borders display fewer BEAF-32 binding sites than maintained and new borders, whereas new borders also show a higher number of Dref binding sites compared to lost borders (Supplemental Fig. S4I). This suggests that a strong but partial depletion of the architectural proteins will first affect the binding at the lost borders. Furthermore, due to the higher number of BEAF-32 and Dref binding sites at new borders compared to lost border, the binding of the architectural proteins (BEAF-32 and Dref) will be maintained at new borders, and thus, lost borders would relocate to the nearby new BEAF-32/Dref-bound regions (Supplemental Fig. S5).

### Combined *Dref* and *BEAF*-*32* knockdown shows an enhanced effect on TAD border distribution

**Figure 2.**
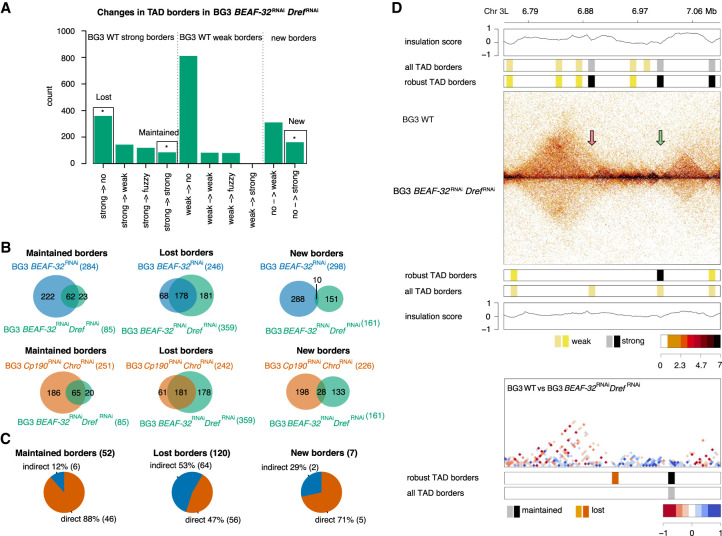
Functional role of Dref in 3D chromatin organization of BG3 cells. (*A*) Classification of robust TAD borders as described in the main text: lost (borders that are strong in WT and completely disappear in the knockdown); maintained (borders that are strong in WT and are maintained strong in the knockdown); and new (borders that appear strong in the knockdown); see Supplemental Figure S3A. (*B*) Overlap of lost, maintained, and new borders in the three knockdowns. (*C*) Number and percentage of maintained, lost, and new borders that have direct binding of BEAF-32, Cp190, and/or Chro (see Supplemental Fig. S4B). We considered common borders between the all three knockdowns (*BEAF-32* single knockdown, *Cp190* and *Chro* double knockdown, and *BEAF-32* and *Dref* double knockdown). (*D*) Examples of genomic regions at DpnII restriction size resolution for *BEAF-32 Dref* double knockdown. We used the same types of plots as in Figure 1E,F.

Dref is a DNA-binding protein that shares a similar binding motif with BEAF-32, meaning that upon depletion of BEAF-32, Dref could potentially replace it at TAD borders. To investigate this, we performed a combinatorial knockdown of *BEAF-32* and *Dref* (Supplemental Fig. S1) followed by in situ Hi-C (Supplemental Tables S1, S2). Again, the combinatorial knockdown resulted in specific and efficient depletion at both mRNA and protein levels and does not affect the cell cycle (Supplemental Fig. S1). In the *BEAF-32 Dref* double knockdown (BG3 *BEAF-32*^RNAi^ *Dref*^RNAi^), we noticed a more pronounced effect in the reorganization of the 3D interaction compared to WT than for BG3 *BEAF-32*^RNAi^ or BG3 *Cp190*^RNAi^ *Chro*^RNAi^ knockdowns compared to WT (Supplemental Fig. S2A–E). In particular, BG3 *BEAF-32*^RNAi^ *Dref*^RNAi^ displayed significantly fewer robust TAD borders (982), of which only one-third are strong (292) (Supplemental Fig. S3A), with the majority of the TAD borders being lost (Fig. 2A,D). There were 50% more TAD borders that were lost in BG3 *BEAF-32*^RNAi^ *Dref*^RNAi^ compared to the single knockdown of BEAF-32 or double knockdown of *Cp190* and *Chro* (Fig. 2B). The difference in insulation between maintained and lost borders increases in the BG3 *BEAF-32*^RNAi^ *Dref*^RNAi^ knockdown (Supplemental Fig. S3B,C). When looking at the maintained borders, only one-third of the borders were maintained in BG3 *BEAF-32*^RNAi^ *Dref*^RNAi^ when compared to BG3 *BEAF-32*^RNAi^ or BG3 *Cp190*^RNAi^ *Chro*^RNAi^ (Fig. 2B). In addition, 161 new borders appear in the BG3 *BEAF-32*^RNAi^ *Dref*^RNAi^ double knockdown (see Fig. 2A,B). The majority of these new borders are movements of borders in the double knockdown compared to the closest WT border (Supplemental Fig. S5). Overall, we found that there is a large overlap between TAD borders that are lost in the three knockdowns and also a large subset of TAD borders that disappear only in the BG3 *BEAF-32*^RNAi^ *Dref*^RNAi^ knockdown, indicating that there is a subset of TAD borders that require Dref for maintenance (Fig. 2B).

To distinguish the direct targets from indirect, we aligned TAD borders with the protein occupancy in WT BG3 cells (see Methods and Supplemental Fig. S4D). The majority of TAD borders that are maintained in BG3 *BEAF-32*^RNAi^ *Dref*^RNAi^ (and also in BG3 *BEAF-32*^RNAi^ and BG3 *Cp190*^RNAi^ *Chro*^RNAi^) (88%) are direct targets of BEAF-32, Cp190, and/or Chro (see Fig. 2C; Supplemental Fig. S4D). However, only half of the lost TAD borders in BG3 *BEAF-32*^RNAi^ *Dref*^RNAi^ (47%) (and also in BG3 *BEAF-32*^RNAi^ and BG3 *Cp190*^RNAi^ *Chro*^RNAi^) are direct targets of the three proteins. Upon single knockdown of BEAF-32 or Cp190, the majority of the maintained TAD borders in BG3 *BEAF-32*^RNAi^ *Dref*^RNAi^ (70%) retain BEAF-32 or Cp190, and most of the lost borders in BG3 *BEAF-32*^RNAi^ *Dref*^RNAi^ (65%) have lost occupancy of these proteins (Supplemental Fig. S4E,F). Similarly to maintained borders, the majority of new borders display binding of BEAF-32, Chro, and/or Cp190. Binding of BEAF-32 or Cp190 is retained at these new borders upon knockdown. Given that the RNAi knockdowns do not completely deplete the architectural proteins (Supplemental Fig. S1D), not all TAD borders would lose the occupancy of these proteins. These results are similar to the ones for the maintained, lost, and new borders common between BG3 *BEAF-32*^RNAi^ and BG3 *Cp190*^RNAi^ *Chro*^RNAi^.

The majority of TAD borders that are lost only in BG3 *BEAF-32*^RNAi^ *Dref*^RNAi^ (and are maintained in BG3 *BEAF-32*^RNAi^ or BG3 *Cp190*^RNAi^ *Chro*^RNAi^) are bound by BEAF-32, Cp190, and/or Chro in WT cells (Supplemental Fig. S4G,H). In addition, these TAD borders that are lost only in BG3 *BEAF-32*^RNAi^ *Dref*^RNAi^ have significantly more binding sites for BEAF-32 but not for Dref (Supplemental Fig. S4I). This suggests that Dref may display redundancy to BEAF-32 by maintaining TAD borders when BEAF-32 is absent. When both architectural proteins are depleted, then these TAD borders that were maintained after *BEAF-32* single knockdown are also lost.

### Reorganization in TADs correlates with changes in gene expression

**Figure 3.**
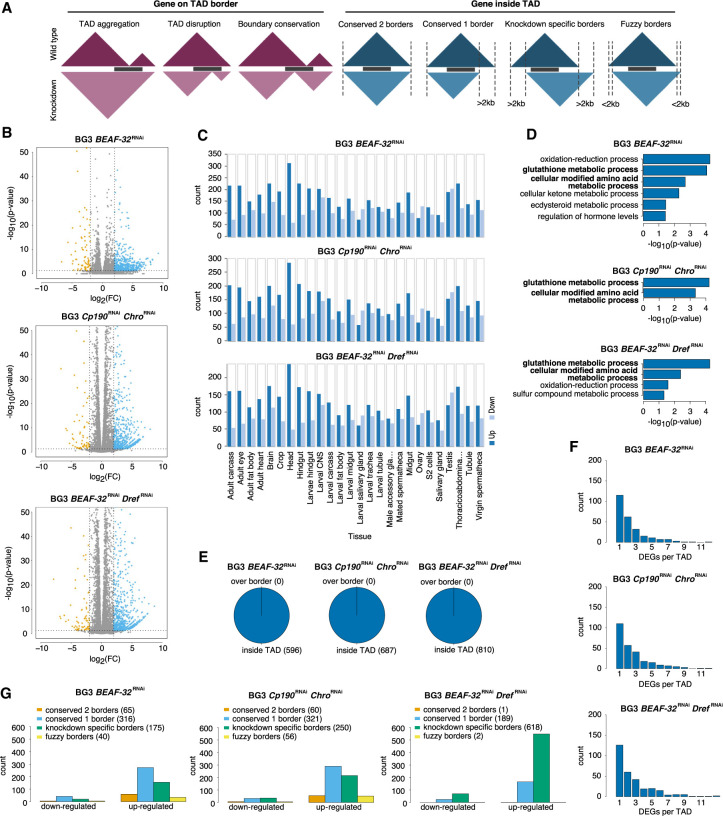
The effects of TAD reorganization on transcription. (*A*) The different cases for position of genes in TADs and how the TADs change in the knockdown (red for cases where the gene spans over TAD borders and blue for the cases where the gene is within the TADs). (*B*–*G*) We consider the case of the three knockdowns separately: *BEAF-32* knockdown, *Cp190 Chro* double knockdown, and *BEAF-32 Dref* double knockdown. (*B*) Volcano plots for the RNA-seq analysis (orange represents down-regulated genes, blue up-regulated, and gray non-DEG) in the three knockdowns. (*C*) FlyAtlas expression data (Chintapalli et al. 2007) for all DEGs in each knockdown. For this analysis, we used FlyMine web server (Lyne et al. 2007). (*D*) GO enrichment analysis of all DEGs in each knockdown using the FlyMine web server (Lyne et al. 2007). Bold terms are the ones common in all three knockdowns. (*E*) The number of differentially expressed genes, where over boarder represents red scenario from *A* and inside TAD represents blue scenario from *A*. (*F*) Histogram with the number of DEGs in TADs (large number of TADs have more than one DEG). (*G*) The number of down-regulated and up-regulated genes in different cases where the gene is within the TADs (orange: both TAD borders are conserved, blue: only one of the TAD border is conserved, green: none of the TAD border is conserved, and yellow: TAD borders are shifted within 2 kb).

Several studies have shown that TAD reorganization leads to changes in transcription that correspond to developmental defects or diseases (Chandra et al. 2015; Lupiáñez et al. 2015; Flavahan et al. 2016; Hnisz et al. 2016; Sun et al. 2018; Kraft et al. 2019). Nevertheless, other studies failed to find a connection between changes in TADs and transcription (Ghavi-Helm et al. 2019; Williamson et al. 2019). Here, instead of disrupting TADs by rearrangements of the DNA at TAD borders, we perturbed a large number of TADs by knocking down architectural proteins and investigated whether that leads to changes in gene expression (Fig. 3A). We found significant changes in gene expression with 598, 688, and 814 differentially expressed genes (DEGs) in BG3 *BEAF-32*^RNAi^, BG3 *Cp190*^RNAi^ *Chro*^RNAi^, and BG3 *BEAF-32*^RNAi^ *Dref*^RNAi^, respectively (Fig. 3B; Supplemental Table S3). The majority of DEGs are up-regulated in the knockdowns compared to WT. We used a FlyAtlas data set (Chintapalli et al. 2007) to investigate if these DEGs are expressed in any particular tissue/cell, and we found that they are mainly expressed in head and brain (Fig. 3C). Furthermore, the DEGs are enriched in glutathione metabolic process and cellular modified amino acid metabolic process Gene Ontology (GO) terms in all three knockdowns (Fig. 3D).

None of these DEGs span over robust TAD borders in either WT or knockdowns (Fig. 3E), and several TADs contain multiple DEGs (Fig. 3F). Figure 3G shows that very few DEGs are in TADs that have both borders conserved in the knockdowns (10.9% in BG3 *BEAF-32*^RNAi^, 8.7% in BG3 *Cp190*^RNAi^ *Chro*^RNAi^, and 0.1% in BG3 *BEAF-32*^RNAi^ *Dref*^RNAi^) (also see Supplemental Fig. S6A). This means that majority of DEGs (at least 89%) are located in TADs where at least one of the borders moves in the knockdowns. There were a large number of DEGs in BG3 *BEAF-32*^RNAi^ *Dref*^RNAi^ where both TAD borders are lost (or move more than 2 kb away), but this could be a consequence of the reduced number of TADs in that knockdown and the corresponding loss of TAD borders. Furthermore, we performed a permutation test and showed that the association of the DEG with reorganized TADs is statistically significant for BG3 *BEAF-32*^RNAi^ and BG3 *BEAF-32*^RNAi^ *Dref*^RNAi^ knockdowns—specifically for TADs in which the borders move more than 2 kb away (Supplemental Fig. S6). Whereas there are many DEGs in BG3 *Cp190*^RNAi^ *Chro*^RNAi^, their association with reorganized TADs is not significant. These changes in border positioning cover several massive rearrangement scenarios, such as significant disruption of WT TADs, aggregation of several WT TADs, or a combination of both. DEGs are randomly distributed inside TADs (no gene spanning over multiple TADs) (Fig. 3E) with no specific localization near or away from TAD borders (see Supplemental Fig. S7). Our results show that mainly large reorganizations of TADs correspond to significant changes in gene expression and explain why previous studies found contradicting results when establishing a link between TADs and gene expression.

### TAD borders are maintained by architectural proteins, divergent transcription, and associated factors

In BG3 *BEAF-32*^RNAi^ and BG3 *Cp190*^RNAi^ *Chro*^RNAi^ knockdowns, we identified two classes of TAD borders: (1) maintained in both knockdowns; and (2) lost in both knockdowns. Given that very few TAD borders are maintained in BG3 *BEAF-32*^RNAi^ *Dref*^RNAi^, whereas the majority are lost, we did not include this in the downstream analysis; that is, the majority of TAD borders that are lost in BG3 *BEAF-32*^RNAi^ and BG3 *Cp190*^RNAi^ *Chro*^RNAi^ are also lost in BG3 *BEAF-32*^RNAi^ *Dref*^RNAi^, but only a few that are maintained in BG3 *BEAF-32*^RNAi^ and BG3 *Cp190*^RNAi^ *Chro*^RNAi^ are also maintained in BG3 *BEAF-32*^RNAi^ *Dref*^RNAi^. Furthermore, we selected maintained and lost TAD borders that display binding in WT cells of BEAF-32, Cp190, or Chro and classified these as direct maintained and lost borders.

**Figure 4.**
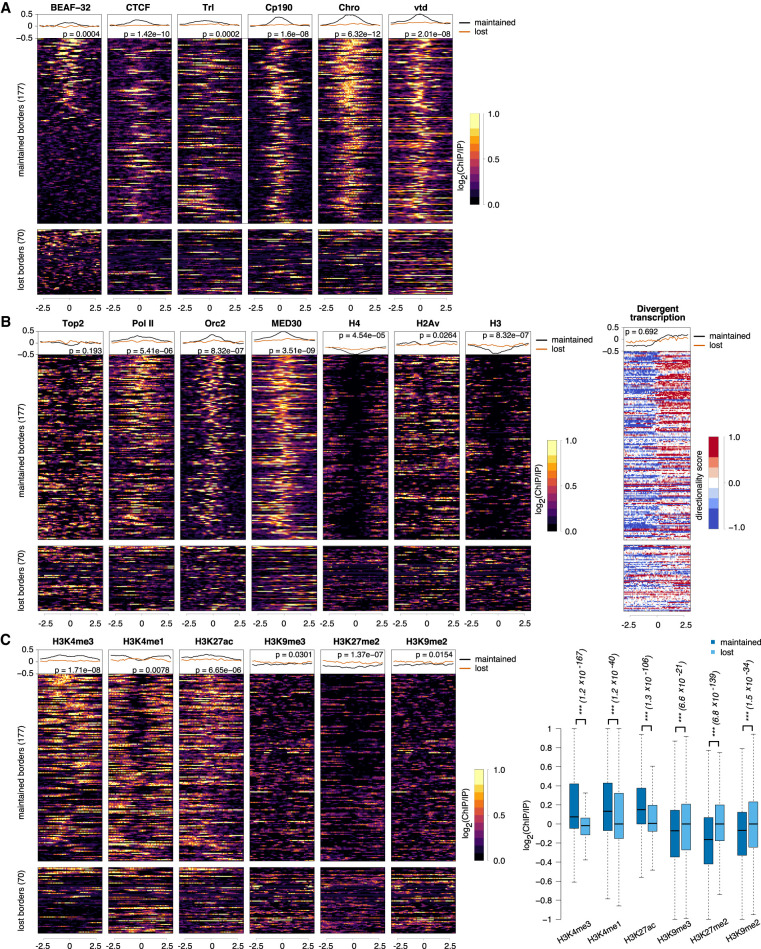
Chromatin feature enrichments at TAD borders. (*A*) Profiles of architectural proteins (BEAF-32, CTCF, Trl, Cp190, Chro, and vtd) around direct maintained and lost TAD borders that were common in *BEAF-32* knockdown and *Cp190 Chro* double knockdown. *Top* lines represent the corresponding average profiles at maintained and lost borders. We also performed the Mann–Whitney *U* test between the average signal at each maintained border and each lost border (corresponding *P*-value added to the plot). (*B*) Profiles of histones (H4, H3, and H2Av), transcription (Pol II, 3′NT-seq, MED30, and Top2), and replication (Orc2) at maintained and lost TAD borders. For nascent transcription, we used two color schemes: orange for transcription on the negative strand and blue for transcription on the positive strand. (*C*) Profiles of histone modifications (H3K4me3, H3K4me1, H3K27ac, H3K9me3, H3K27me2, and H3K9me2) at maintained and lost TAD borders. There is depletion of signal in the middle of the histone modifications heat maps, which can be explained by the depletion of histones in those regions (see *B*).

To determine the chromatin and epigenetic mechanisms present at maintained and lost borders, we analyzed the presence of key factors (such as architectural proteins, transcription, replication, and accessibility related complexes). CTCF was partially present at the maintained borders (approximately at half of the borders), but there was a strong enrichment of cohesin at a majority of maintained borders (vtd [also known as Rad21], Nipped-B, and SMC1) and Trl (Fig. 4A; Supplemental Figs. S4A, S8A). Furthermore, the maintained borders were enriched with Pol II, Mediator complex (MED30 and MED1), and Orc2. Significantly lower histone levels (H4/H3/H1) at maintained borders indicated the presence of highly accessible DNA (Fig. 4B; Supplemental Fig. S8B). Noticeably, there is also strong divergent transcription at the maintained borders (Fig. 4B). This strong divergent transcription at maintained TAD borders coupled with the lack of enrichment for Top2 (Fig. 4B; Supplemental Fig. S8B) indicates a potential role for supercoiling at these borders.

The RNA-seq signal around maintained and lost TAD borders does not show noticeable changes in the two knockdowns (Supplemental Fig. S8B). At maintained borders, given that there are negligible changes in gene expression, these results were expected (see Fig. 3). Nevertheless, given the large number of differentially expressed genes associated with reorganized TADs (see Fig. 3), one could expect a change in the RNA-seq signal at lost TAD borders in the two knockdowns (BG3 *BEAF-32*^RNAi^ and BG3 *Cp190*^RNAi^ *Chro*^RNAi^). Because the differentially expressed genes are randomly located inside the TAD (Supplemental Fig. S7), loss of TAD borders will often correlate with changes in gene expression at a larger distance from the TAD border, and this cannot be captured in the analysis in the vicinity of TAD borders (Fig. 4B; Supplemental Fig. S8B). Nevertheless, Figure 3 and Supplemental Figure S6 confirmed that TADs with lost or new borders harbor more DEGs than TADs with both borders maintained or with fuzzy borders in the knockdowns.

In contrast, at lost TAD borders (in BG3 *BEAF-32*^RNAi^ and BG3 *Cp190*^RNAi^ *Chro*^RNAi^), there is less DNA accessibility and transcription, indicating that these borders are in a repressed chromatin state (Fig. 4B; Supplemental Fig. S8B).

### Maintained borders are associated with active promoters and enhancers, whereas lost borders are located in heterochromatin

Regulatory regions in the DNA can be defined by the presence of specific histone marks (Kharchenko et al. 2011). Transcription has also been shown to be strongly implicated in the maintenance and formation of TADs (Li et al. 2015; Ulianov et al. 2016; Rowley et al. 2017). The presence of Pol II and nascent transcription at maintained borders and their absence from lost borders indicate the existence of two classes of TAD borders in *Drosophila*, active and repressed borders, which display different mechanisms of maintenance. A similar classification into active and repressed domains in *Drosophila* has been previously proposed (Ogiyama et al. 2018; Szabo et al. 2018). We investigated the presence of histone modifications to further dissect the potential factors and mechanisms that would be responsible for the maintenance of the TAD borders. We found that H3K4me3 (active promoter mark) and H3K4me1 (enhancer mark) together with H3K27ac were enriched at all maintained borders (Fig. 4C; Supplemental Fig. S8C). Depletion of BEAF-32 from promoters and enhancers is not sufficient to result in the loss of these TAD borders, which indicates the presence of a redundant mechanism with a different protein(s).

We observed strong enrichment of mof (involved in maintenance of H4K16ac), Kdm2 (H3K36me3 demethylase), Iswi and E(bx) (also known as NURF301) (nucleosome sliding), and wds (involved in maintenance of H3K4me3) preferentially at maintained borders (Supplemental Fig. S9). E(bx) was shown to colocalize together with Dref and Cp190 (Kwon et al. 2016), which explains its enhanced level at the maintained TAD borders.

The lost borders were moderately enriched in H3K9me2, H3K9me3, and H3K27me2 (signatures for heterochromatin and Polycomb) and depleted in active modifications (e.g., H3K4me3), suggesting a plausible association of these borders with heterochromatin regions (Fig. 4C; Supplemental Fig. S8C). As we observed association of lost borders with heterochromatin and Polycomb, we further dissected and analyzed the Polycomb complexes in detail at all borders. However, we did not observe enrichment of any Polycomb subcomplexes (Pc or dRING) at lost borders in the two knockdowns (Supplemental Fig. S9). Nevertheless, we did find enrichment of Su(var)3-9 and Su(var)2-HP2 (also known as HP2), which explains the enrichment of heterochromatin at lost TAD borders in the two knockdowns (Supplemental Fig. S9). Note that, in *Drosophila*, Su(var)3-9 was previously reported to have a role in maintenance of TADs located in heterochromatin (Saha et al. 2020).

Whereas we observed heterochromatic signatures at the lost borders (Fig. 4C; Supplemental Fig. S8C), previous research reported that TAD borders are mostly composed of euchromatin (Sexton et al. 2012; Ulianov et al. 2016; Hug et al. 2017; Ramírez et al. 2018; Chathoth and Zabet 2019). Using a chromatin state map in BG3 cells (Skalska et al. 2015), we investigated the chromatin states associated with maintained, new, and lost TAD borders in each knockdown. Our results confirm that indeed maintained, lost, and new borders are enriched in enhancer and active TSS chromatin states (Supplemental Fig. S10A–C) and are depleted in heterochromatin (Supplemental Fig. S10A–C). In addition, lost borders also display partial enrichment in Polycomb state. This apparent difference in results at lost TAD borders can be explained by the fact that the analysis in Supplemental Figure S10A through C, is performed on TAD borders at a base pair resolution, whereas the analysis in Figure 4 and Supplemental Figures S8 and S9 was performed over a 5-kb region. When considering the same 5-kb regions as in Figure 4 and Supplemental Figures S8 and S9, one can observe an enrichment for Polycomb state and a lower enrichment for heterochromatin in euchromatin at lost TAD borders (Supplemental Fig. S10D). This means that whereas the majority of borders are enriched in enhancers or active TSSs, maintained borders are located in euchromatin and lost borders in euchromatin islands in heterochromatin.

Compared to maintained and new borders, lost TAD borders are also enriched in clusters of noncoding regulatory elements that display extreme levels of sequence conservation (gene regulatory blocks, GRBs) (Supplemental Fig. S10E; Harmston et al. 2017). It is worthwhile noting that GRBs are enriched in transcriptionally silent and Polycomb regions (Harmston et al. 2017), further supporting the localization of the lost TAD borders in the repressed chromatin state.

One possibility is that lost borders, although euchromatic, display higher levels of Pol II pausing. Using the Pol II pausing index definition from Ramírez et al. (2018) (see Methods), we found only negligible differences in Pol II pausing for genes located within 5 kb windows around maintained, lost, and new borders (Supplemental Fig. S10F). This indicates that Pol II pausing does not differentially affect maintained or lost borders.

### A large proportion of maintained TAD borders in the knockdowns are also present in Kc167 cells and harbor housekeeping genes

Previously, we showed that Kc167 cells display more short-range interactions and fewer long-range contacts when compared to BG3 cells, which was true also after down-sampling to control for library size differences (Chathoth and Zabet 2019). Given that the three knockdowns we analyzed here display increased numbers of short-range contacts and reduced numbers of long-range contacts compared to WT BG3 cells (Supplemental Fig. S2A–D), this raises the question of how the 3D organization of these knockdowns differs when compared to Kc167 cells. Our results show that there are significantly more short-range interactions and fewer long-range interactions in Kc167 cells compared to BG3 *BEAF-32*^RNAi^, BG3 *Cp190*^RNAi^ *Chro*^RNAi^, and BG3 *BEAF-32*^RNAi^ *Dref*^RNAi^ (Supplemental Fig. S11A). To further investigate the similarities between the BG3 *BEAF-32*^RNAi^, BG3 *Cp190*^RNAi^ *Chro*^RNAi^, and BG3 *BEAF-32*^RNAi^ *Dref*^RNAi^ and Kc167 cells, we compared the maintained, lost, and new robust TAD borders in the knockdowns with the robust TAD borders in Kc167 cells. Approximately half of the maintained TAD borders in the three knockdowns are also strong TAD borders in Kc167 cells, but this decreases to <20% for lost and new borders (Supplemental Fig. S11B). This is true when comparing to both similar size (Chathoth and Zabet 2019) or significantly larger (Eagen et al. 2017) Hi-C libraries in Kc167 cells. This indicates that nearly half of the maintained borders are housekeeping TAD borders, whereas the majority of lost borders are BG3-specific. The majority of genes present at the TAD borders conserved between Kc167 WT, BG3 WT, BG3 *BEAF-32*^RNAi^, and BG3 *Cp190*^RNAi^ *Chro*^RNAi^ (176 out of 181) are housekeeping genes (Supplemental Table S4; Methods). This together with the fact that maintained TAD borders display divergent transcription (Fig. 4B) indicates that the majority of conserved TAD borders are divergently oriented housekeeping genes.

### Majority of chromatin loops in *Drosophila* are controlled by Mediator complex, Chro, and Cp190

**Figure 5.**
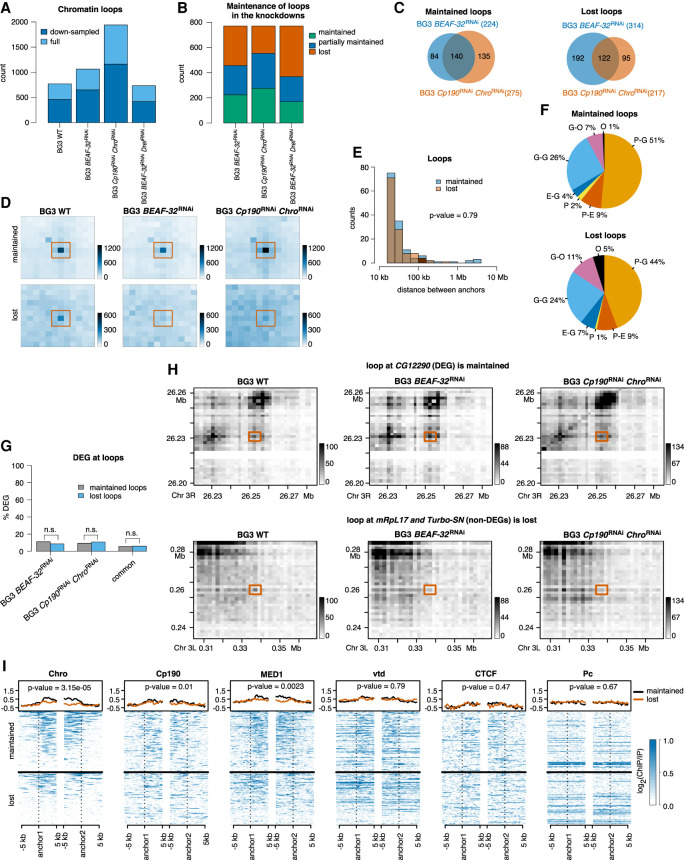
Chromatin loops. (*A*) Number of loops detected in WT and the three knockdowns. Dark blue represents the loops that are detected in both full and down-sampled (where we randomly removed 20% of the reads) data sets, and light blue represents loops detected only in the full data set. (*B*) Number of loops in the three knockdowns that maintain both of the anchors (maintained), only one of them (partially maintained), or lose both anchors (lost). (*C*) Overlap of loops maintained or lost between WT and *BEAF-32* knockdown and between WT and *Cp190 Chro* double knockdown. We classify the commonly maintained borders in the two knockdowns as maintained and the commonly lost borders as lost. (*D*) Aggregate peak analysis (APA) using Juicer (Durand et al. 2016) over the maintained (*top*) and lost (*bottom*) chromatin loops at 2-kb resolution. (*E*) Size of the maintained and lost chromatin loops. We performed a Mann–Whitney *U* test, which confirmed that the two distributions are not different. (*F*) Annotation of maintained and lost loops with respect to the features they connect: (P) promoters (up to 1 kb upstream of TSS), (E) enhancers, (G) genes, and (O) others. We used STARR-seq for enhancer annotation (Yanez-Cuna et al. 2014). (*G*) Percentage of genes that are differentially expressed and are associated with maintained and lost chromatin loops. We selected genes that have their promoter (up to 1 kb upstream of TSS) located at one of the anchors of the chromatin loops. There is no statistically significant difference between DEG at maintained and lost loops (Fisher's exact test; *P*-value 0.37, 1.0, and 1.0). (*H*) Contact matrices plots of a maintained (*top*) and a lost (*bottom*) loop. These maps were constructed with diffHic (Lun and Smyth 2015) at 2-kb resolution (the same used to detect loops) and contain 30 bins. Dark color represents more contact. We scaled the pallet in the two knockdowns to account for library size differences. (*I*) Enrichment of architectural proteins and transcription-related factors at maintained and lost loops (Chro, Cp190, MED1, vtd, CTCF, and Pc). We performed a Mann–Whitney *U* test of the mean signal at maintained and lost loops (see corresponding *P*-values).

Chromatin loops represent enriched long-range 3D interactions and have been identified as important features in 3D chromatin organization. In *Drosophila*, only a small number of loops have been detected (Eagen et al. 2017; Chathoth and Zabet 2019). We identified loops in WT BG3 cells and in the three knockdowns and observed an increase in the number of loops in two knockdowns (BG3 *BEAF-32*^RNAi^ and BG3 *Cp190*^RNAi^ *Chro*^RNAi^) (Fig. 5A). This could be attributed to the difference in sequencing depth between the different samples, and when we analyzed 20% down-sampled libraries, we observed a reduction in the number of chromatin loops detected (Fig. 5A). Of the 770 loops that were detected in WT cells, in each knockdown, approximately 200 are maintained and 300 maintain only one anchor in the same position (Fig. 5B). We classified 140 loops that are maintained in both *BEAF-32*^RNAi^ and BG3 *Cp190*^RNAi^ *Chro*^RNAi^ knockdowns as maintained loops and 122 that are lost in both BG3 *BEAF-32*^RNAi^ and BG3 *Cp190*^RNAi^ *Chro*^RNAi^ knockdowns as lost loops (Fig. 5C). By focusing only on the common maintained or lost borders between the two knockdowns (BG3 *BEAF-32*^RNAi^ and BG3 *Cp190*^RNAi^ *Chro*^RNAi^) that display different library sizes, we ensure that library size differences are not influencing the downstream analysis. Figure 5D confirms that the strong level of interactions is maintained in the two knockdowns at maintained chromatin loops, but this is not the case at lost loops. We also found that there is no statistically significant difference in the size of the lost and maintained chromatin loops (Fig. 5E).

Of these loops, 68%–77% connect different parts of genes to each other (e.g., promoters, intronic enhancers, or 3′ UTRs) or to other genes (e.g., intronic enhancer controlling a distal promoter or promoter hubs) (Fig. 5F), indicating that they are involved in the formation of gene domains (Rowley et al. 2019). Only 9% of the maintained and lost loops are promoter-enhancer loops (Fig. 5F), which indicates that this mechanism is less prevalent in *Drosophila* than previously proposed in mammalian systems (Noordermeer et al. 2014). When we select all genes that have their promoter located at one of the anchors of the loops, we found that only a small subset of genes (<10%) located at the lost or maintained loops display differential expression in the two knockdowns, and this is true even when using a less stringent threshold to call differentially expressed genes (log_2_FC threshold of 1.0) (Fig. 5G; Supplemental Fig. S12). Furthermore, there is no statistically significant difference in the number of DEGs at maintained and lost loops (Fig. 5G). For example, a chromatin loop can be maintained in the two knockdowns, whereas the target gene is differentially expressed (top panel in Fig. 5H). Conversely, a lost chromatin loop can lead to no changes in gene expression of the target genes (bottom panel in Fig. 5H). Thus, our results support a model where the presence or absence of a chromatin loop does not necessarily lead to regulation of the target gene.

Chro and Cp190 are known to be involved in long-range interactions (Vogelmann et al. 2014), but previous research identified the enrichment of Polycomb at *Drosophila* loops (Eagen et al. 2017). We found that both maintained and lost chromatin loops display high levels of BEAF-32 together with Chro and/or Cp190 at both anchors in WT cells (Fig. 5I; Supplemental Fig. S13A); that is, 92% of maintained and 84% of lost loops have binding of BEAF-32, Cp190, and/or Chro (Supplemental Fig. S13B). The maintained loops display higher levels of Chro at the anchors compared to lost loops, suggesting that the depletion of Chro (in BG3 *Cp190*^RNAi^ *Chro*^RNAi^) or blocking of its recruitment (in BG3 *BEAF-32*^RNAi^) is not sufficient to affect the maintained loops. In addition, 60% of lost loops lose binding of BEAF-32 and/or Cp190 upon their knockdown (Supplemental Fig. S13C,D), thus providing support that these loops are lost as a direct consequence of the depletion of the architectural proteins in our knockdowns. Nevertheless, approximately half of the maintained loops lose BEAF-32 and/or Cp190 upon knockdown (Supplemental Fig. S13C,D), which suggests that Chro is recruited by additional proteins at maintained loops or that other factors could help maintain these loops (Supplemental Fig. S13A). We observed an enrichment of MED1 at the anchors of maintained and lost loops but also enrichment of CTCF and cohesin subunit vtd. The majority of chromatin loops in our data set are located near a MED1 ChIP peak (Supplemental Fig. S13A), indicating that Mediator complex would be more important for chromatin loops in *Drosophila*. We also observed a small number of loops with enrichment of Polycomb peaks near their anchors (Fig. 5; Supplemental Fig. S13), but this is less pronounced than in the case of Mediator complex.

### *BEAF-32, Cp190, Chro*, and *Dref* knockdowns do not affect A/B compartments

The checkerboard pattern seen on Hi-C maps led to the identification of A and B compartments which mark active and inactive regions of chromatin (Lieberman-Aiden et al. 2009). A/B compartments were also identified in *Drosophila* (Rowley et al. 2017) using 10-kb bins, and we showed that compartmentalization changes between cell lines (Chathoth and Zabet 2019). The current working model assumes that compartments harbor several TADs and they display different mechanisms for maintenance compared to TADs. To investigate if the changes in the TADs lead to changes in the A/B compartmentalization of the genome, we computed the A/B compartments at 10-kb resolution in the three knockdowns (see Methods). Our results confirm that there are negligible changes in the proportion of the genome that is in the A or B compartments in all knockdowns (Supplemental Fig. S14A). Nevertheless, we identified some switching between the A and B compartments (<5%) (Supplemental Fig. S14B). When we zoomed in, we observed that the majority of these compartments are robust and consistent in the WT and knockdowns (Supplemental Fig. S14C). One interesting observation is that there is some rare local spreading of the B compartment (heterochromatin) into the A compartment (euchromatin) (e.g., yellow stripe in Supplemental Fig. S14C).

Saddle plots confirm that regions belonging to the same compartments (lower right corner A-A interactions and upper left corner B-B interactions) are enriched in interactions, whereas regions belonging to different compartments (lower left corner A-B interactions and upper right corner B-A interactions) are depleted in interactions (Supplemental Fig. S14D). Compartments strengths are similar, with only a small decrease for *BEAF-32* single knockdown. This confirms that BEAF-32, Cp190, Chro, and Dref have little effect on compartmentalization. Altogether, our results indicate that organization of compartments in *Drosophila* is independent of the organization of TADs.

When investigating in which compartments TAD borders are localized, we found that most of the maintained borders are localized in the A compartment, whereas most of the lost or new borders are localized in the B compartment (Supplemental Fig. S15A). This is not surprising, because most of the lost borders are located in repressed chromatin, whereas the maintained ones are in active chromatin.

The majority of compartments that switch do not harbor any DEGs, even when using a lower threshold to call differential gene expression (log_2_FC threshold of 1) (Supplemental Fig. S15B). Furthermore, the fact that a compartment contains DEGs does not mean that all genes in that compartment change expression in the same direction (either up-regulated or down-regulated). For example, spreading of the B compartment in Supplemental Figure S14C corresponds to three genes displaying different behaviors: *ine* gene is down-regulated; *Dp* is up-regulated; and *FIG4* maintains expression in all three knockdowns (all three genes are located within the yellow stripe in Supplemental Fig. S14C). The relationship between changes in gene expression and compartment switching is complex, and often compartment switching cannot be explained by a majority of genes changing expression in the same direction. Note that RNA-seq libraries capture only poly(A) transcripts and do not include other transcripts such as eRNAs or lncRNAs, which could potentially contribute to compartment switching.

## Discussion

The enrichment of architectural proteins at TAD borders raises the question of whether they have a functional role in TAD organization or whether their colocalization with borders is correlative in nature. In mammalian systems, depletion of CTCF or cohesin disrupts TADs (Zuin et al. 2014; Nora et al. 2017; Schwarzer et al. 2017). In *Drosophila*, several architecture proteins (including BEAF-32, Chro, and Cp190) are enriched at TAD borders, but their functional role at TAD borders has not previously been investigated (Van Bortle et al. 2014; El-Sharnouby et al. 2017; Hug et al. 2017; Ramírez et al. 2018; Chathoth and Zabet 2019; Matthews and White 2019). Our results confirm that the architectural proteins are essential for TAD borders and their depletion results in reorganization of TADs. In particular, we found that TAD borders mainly found in mostly silenced regions of the genome (located in the B compartment and displaying moderate enrichment of heterochromatin and GRBs and depletion of active histone modifications) are lost upon depletion of BEAF-32 or Cp190 and Chro. Cp190 and Chro cannot bind independently to DNA, but are recruited, mainly by BEAF-32 (Vogelmann et al. 2014). The majority of the lost borders are common between the *BEAF-32* knockdown and *Cp190* and *Chro* double knockdown, but there are also borders that are specific to each knockdown.

Furthermore, we identify a subset of TAD borders that are not affected by the depletion of BEAF-32, Cp190, and Chro. These borders are enriched in cohesin and Mediator complex and also in CTCF and Trithorax-group (fs(1)h, E(bx), Iswi, mod[mdg4], ash1, and Trl). This supports a model where several complexes are redundant and can compensate for the loss of BEAF-32, Cp190, or Chro. However, 70% of TAD borders that are maintained have retained binding of BEAF-32 and/or Cp190 upon the depletion of these architectural proteins (see Supplemental Fig. S4B,C).

Finally, Dref shares a similar binding motif to BEAF-32, which suggests that it could potentially replace it following *BEAF-32* knockdown. Our *BEAF-32 Dref* double knockdown results in a larger number of TAD borders being lost, supporting the model in which Dref compensates the loss of BEAF-32. The borders that are specifically lost in the *BEAF-32* and *Dref* double knockdown are borders displaying binding of BEAF-32, Cp190, and/or Chro, and thus Dref would provide redundancy for partial loss of BEAF-32.

To investigate that the effects we observe in 3D chromatin organization are not a reflection of cell cycle arrest (Gibcus et al. 2018; Ramírez et al. 2018) but are due to the knockdown of architectural proteins, we have performed a FACS analysis. This showed that none of our knockdowns lead to changes in the cell cycle progression (Supplemental Fig. S1E), thus confirming that the changes in 3D chromatin organization are not caused by cell cycle arrest.

Altogether, our results confirm that, whereas the majority of TAD borders are enriched in enhancers or active TSSs, there are two classes of TAD borders: (1) TAD borders located in euchromatin; and (2) TAD borders located in heterochromatin. Whereas the former are maintained upon depletion of BEAF-32, Cp190, and Chro, the latter are lost. This classification of TAD borders is additionally supported by the preferential localization of maintained borders in the A compartment (active chromatin) and of lost and new borders in the B compartment (repressive chromatin) (see Supplemental Fig. S15A).

The enrichment of divergent transcription at TAD borders that are maintained in the two knockdowns, when coupled with the lack of enrichment for Top2 at these borders, possibly indicates that negative supercoiling accumulates at these TAD borders, which may be due to active transcription. This negative supercoiling is not relaxed due to lack of Top2. When negative supercoiling accumulates at maintained borders, positive supercoiling may accumulate inside TADs, which indicates a role for supercoiling in TAD borders (Benedetti et al. 2017; Björkegren and Baranello 2018). Due to a lack of divergent transcription at lost borders, there is no accumulation of negative supercoiling at these TAD borders and the reduced levels of Top2 will not have an effect. Nevertheless, the role of supercoiling at TAD borders requires further investigation with direct measurement of supercoiling.

The detection of the maintained and lost TAD borders was performed following a robust analysis using five filtering steps to select lost or maintain TAD borders. First, we detected TAD borders using two thresholds: a stringent threshold for strong borders; and a less stringent one for weaker borders. Second, to account for differences in Hi-C libraries, for strong and weak borders separately, we selected robust TAD borders as borders that are still detected after the Hi-C libraries are down-sampled by 20%. Third, for each knockdown, we defined lost borders to be those strong robust TAD borders in WT that have not been identified as strong or weak borders in the knockdowns. Similarly, we defined maintained borders as strong robust TAD borders in WT that are detected as strong robust TAD borders in the knockdowns. Fourth, our analysis focused on the maintained and lost borders that are common between the *BEAF-32* single knockdown and *Cp190* and *Chro* double knockdown. Fifth, we selected as direct maintained and lost borders the common maintained and lost TAD borders that displayed a BEAF-32, Cp190, and/or Chro ChIP peak in their vicinity. Altogether, this analysis supports that our results are robust. It is worthwhile noting that many TAD reorganizations are observed because of the noisy nature of Hi-C data. To account for this, we focused our analysis on the higher confidence TAD border changes. Thus, in our analysis, the number of TAD borders that are robustly detected as maintained or lost is a small subset (∼25% of the strong borders) compared to the TAD borders that are fuzzy or weakened.

### TAD reorganization and transcription

We identified between approximately 600 and 800 differentially expressed genes in the three knockdowns, and the majority of those are located within TADs that lost one or both borders or shifted the position of the borders (more than 89%). We also found that there are more statistically significant DEGs than expected by chance in reorganized TADs; however, this is mainly the case when TAD borders move more than 2 kb away from their WT position (Supplemental Fig. S6B). This indicates that usually strong TAD reorganization is coupled with significant changes in gene expression. Nevertheless, there are also examples where discrete changes in TAD borders correspond to changes in gene expression (Mateo et al. 2019; Arzate-Mejía et al. 2020). For the *Cp190* and *Chro* double knockdown, we did not see a statistically significant association between DEGs in reorganized TADs (Supplemental Fig. S6B). This can be explained by the fact that these two proteins are also recruited to the DNA by other proteins that would not be involved in TAD border organization (Schwartz et al. 2012). In this case, a subset of DEGs in the *Cp190* and *Chro* double knockdown are not associated with reorganization of TADs and, thus, the statistical significance of association of DEG and reorganization of TADs is reduced.

We also observed more up-regulated genes than down-regulated, which suggests that TADs have a role in maintaining a repressed state of chromatin. Down-regulation of genes in these knockdowns can be explained by the loss of TAD borders in heterochromatin. Previous work in *Drosophila* did not identify any connection between changes in TADs and changes in gene expression (Ghavi-Helm et al. 2019). These contradicting results can be explained by the stronger reorganization of the TADs in our knockdowns compared to the TADs reorganization observed on the balancer chromosomes. Recently, it was shown that there are significant changes in gene expression corresponding to reorganization of TADs in human cancers, but only 14% of changes in TAD organization result in strong changes in gene expression (more than twofold) (Akdemir et al. 2020). Our findings are consistent with these results and emphasize that the functional role of TAD organization is conserved between species.

One question that is still unanswered is whether the changes in gene expression are caused by the changes in TAD organization or whether depletion of architectural proteins affects transcription, causing the observed changes in TAD organization. Previous studies showed that TADs appear together with transcription activation in the *Drosophila* zygote, indicating a functional role of transcription in TAD formation, but blocking transcription elongation only marginally affects TADs in the *Drosophila* embryo (Hug et al. 2017). Furthermore, a 10- to 20-fold activation of transcription using the CRISPR-Cas9 system in mouse neuronal progenitor cells was not sufficient to induce TAD boundary formation (Bonev et al. 2017). Results from these alternative approaches suggest that changes in gene expression do not lead to reorganization of TADs, but further work to confirm this is needed.

### Chromatin loops and gene regulation

Our analysis revealed that the chromatin loops in *Drosophila* can be classified into three large classes: (1) BEAF-32 with Chro and/or Cp190; (2) Mediator complex; and (3) Polycomb (Fig. 5D; Supplemental Fig. S13). Previous work reported that chromatin loops in *Drosophila* are controlled by Polycomb (Eagen et al. 2017), but our results show that Polycomb loops are just a small subset compared to Chro/Cp190 and Mediator complex loops. Depleting Chro/Cp190 or BEAF-32 (protein that recruits Chro/Cp190 to DNA) results in the loss of loops, mainly those loops displaying weaker Chro/Cp190 enrichment, suggesting concentration-dependent control. Chro and Cp190 were shown to be involved in long-range interactions in *Drosophila* (Vogelmann et al. 2014), and our results confirm that the majority of chromatin loops in *Drosophila* are controlled by these proteins. We also found enrichment of cohesin and CTCF at chromatin loops, indicating that they might have a role in chromatin loop formation in *Drosophila* (Rao et al. 2014; Rhodes et al. 2020). In particular, half of maintained loops lose BEAF-32 and/or Cp190 binding upon knockdown, indicating that CTCF and cohesin could play a role in the maintenance of these loops.

Some interactions between specific DNA regions identified in the contact maps are shown to arise from promoter-enhancer loops (a chromatin loop having an enhancer at one end and a promoter at the other) (Noordermeer et al. 2014). In *Drosophila* BG3 cells, we found that only 10% of the chromatin loops are promoter-enhancer loops, and one possible explanation for this is that the annotation of enhancers is not comprehensive (Muerdter et al. 2018). Even if this is the case, only approximately half of the loops have promoters at one end, indicating that majority of interactions are not regulatory in nature (Sanyal et al. 2012; Javierre et al. 2016; Zheng et al. 2019). Furthermore, when a promoter has a 3D contact with a regulatory sequence, <10% of genes display differential expression when the contact is lost, but the same is true at maintained loops. This suggests that the presence of chromatin loops would not be essential for controlling gene transcription in the majority of cases (Atlasi et al. 2019; Rhodes et al. 2020; Espinola et al. 2021; Ing-Simmons et al. 2021).

## Methods

### Cell culture and knockdown

*Drosophila* BG3 cells were cultured at 25°C in Schneider's insect medium (Sigma-Aldrich), supplemented with 10% FBS (Labtech), 10 mg/L insulin (Sigma-Aldrich I9278) and the antibiotic Pen-Strep. Primer sequences for Cp190, Chro, and BEAF-32 dsRNAi were obtained from the *Drosophila* RNAi Screening Center database (https://www.flyrnai.org/up-torr/) (see Supplemental Table S5). The primers with T7 promoter sequence were used to amplify the IVT templates from wild-type genomic DNA using a Dream Taq DNA Polymerase kit (Thermo Fisher Scientific EP0703). The PCR products were checked by electrophoresis and purified using a FastGene PCR Purification kit (Fastgene). The purified PCR products were then used as templates to synthesis dsRNA using the MEGAscript T7 kit (Invitrogen AM1334) according to the manufacturer's recommendations. The BG3 cells were transfected with 50 µg of dsRNA using FuGENE (Promega) according to the manufacturer's protocol. Cells were harvested after 72 h and processed for downstream experiments accordingly.

### Western blot

Cells were pelleted, washed in PBS, and resuspended in SDS PAGE loading buffer, at a concentration of 40,000 cells per µL, sonicated, and boiled for 4 min. Five microliters of lysate were loaded on a 10% polyacrylamide gel. The proteins were transferred onto nitrocellulose and saturated 1 h with 5% skimmed milk (or 1% BSA for the Chro antibody) in PBS Tween 0.1%. The blots were incubated overnight with anti-BEAF-32 (Blanton et al. 2003) (1/200), anti-Chro (Rath et al. 2004) (1/200), anti-Cp190 (Whitfield et al. 1988) (1/5000), or anti-Dref (Hirose et al. 1996) (1/5000). Secondary antibodies (peroxidase anti-rabbit for Cp190 and Dref and peroxidase anti-mouse for BEAF and Chro) were incubated at a 1/10,000 dilution. They were visualized with Pierce ECL western blotting substrate using the Fujifilm LAS4000 gel imaging system. Anti-BEAF-32 (AB_1553420) and anti-Chro (12H9-4A2; AB_2721936) were purchased from Developmental Studies Hybridoma Bank, and anti-Cp190 (Whitfield et al. 1988) and anti-Dref (Hirose et al. 1996) were kindly provided by Dr. Rob White and Dr. Professor Masa Yamaguchi, respectively.

### FACS

Cells were pelleted, washed in PBS, and resuspended in 50% ethanol in PBS and stored until analysis at 4°C. On the day of the analysis, cells were pelleted, washed in PBS, and resuspended in FACS PI buffer (PBS, 01% Triton X-100, 100 µg/mL RNase, and 50 µg/mL propidium iodide) at a concentration of 10^6^ cells/mL. The cell cycle profile was analyzed with the Guava easycyte HT flow cytometer using the Incyte software and FlowJo. For each sample, 15,000 cells were analyzed.

### In situ Hi-C protocol

Hi-C libraries were generated from 10 million cells by following the in situ Hi-C protocol as mentioned in Chathoth and Zabet (2019). Briefly, crosslinked cells were lysed, and the genome was digested using DpnII (NEB) overnight. The overhangs were filled with Biotin-16-dATP (Jena Bioscience) followed by ligation and de-crosslinking with Proteinase K digestion. The sample was further sonicated using Bioruptor. Biotinylated DNA was pulled down using Dynabeads MyOne Streptavidin T1 beads (Invitrogen 65602). Selected biotinylated DNA fragments ranging from 200 to 500 bp were then ligated with Illumina adaptors (NEB). The libraries obtained from biological replicates were multiplexed and further sequenced at the Oxford Genomics Centre and Edinburgh Genomics (Genepool) using HiSeq 4000.

### Hi-C analysis

Each pair of the PE reads was aligned separately to the *Drosophila melanogaster* (dm6) genome (Adams et al. 2000; dos Santos et al. 2015) using BWA-MEM (Li and Durbin 2010) (with options -t 20 -A1 -B4 -E50 -L0). HiCExplorer was used to build and correct the contact matrices and detect TADs and enriched contacts (Ramírez et al. 2018). The contact matrices were built using the DpnII restriction sites. We also used 100-kb bins for plotting Supplemental Figure S2 only and 10-kb for compartments (Rowley et al. 2017). Using a minimum allowed distance between restriction sites of 150 bp and a maximum distance of 1000 bp, we obtained a matrix with 217,638 bins with a median width of 529 bp. After filtering, we obtained between 18 M and 65 M valid pairs (see Supplemental Table S1). Note that the number of reads and valid pairs used in this study are within values successfully used for previous work in *Drosophila* cells to detect TADs, chromatin loops, and compartments; for example, Cubeñas-Potts et al. (2017), Ramírez et al. (2018), and Chathoth and Zabet (2019). In addition, we also showed that these libraries are robust to down-sampling (Supplemental Fig. S3A; Chathoth and Zabet 2019). The matrices were corrected using the thresholds in Supplemental Table S2, where values were selected from the diagnostic plots (Supplemental Fig. S16). By using the corrected contact matrices, we detected TADs of at least 5-kb width using a *P*-value threshold of 0.01, a minimum threshold of the difference between the TAD-separation score of 0.04, and FDR correction for multiple testing (‐‐step 2000, ‐‐minBoundaryDistance 5000 ‐‐pvalue 0.01 ‐‐delta 0.04 ‐‐correctForMultipleTesting fdr). We selected these parameters to ensure that we recover a similar number of TADs as previously reported (Chathoth and Zabet 2019). Finally, we called strong TAD borders using a stringent value of the threshold of the difference between the TAD separation score of 0.08. This value ensured that we retrieved the strongest half of TADs. The enriched contacts were extracted with HiCExplorer using the observed/expected ratio method.

### Chromatin loops

Chromatin loops were called with the HiCCUPS tool from the Juicer software suite (Durand et al. 2016) on all knockdowns as done previously (Chathoth and Zabet 2019). Loops were called using a 2-kb resolution, 0.05 FDR, Knight-Ruiz normalization, a window of 10, peak width of 5, thresholds for merging loops of 0.02, 1.5, 1.75, 2, and distance to merge peaks of 20 kb (-k KR -r 2000 -f 0.05 -p 5 -i 10 -t 0.02,1.5,1.75,2 -d 20000).

### Compartments

Compartments were called as described in Lieberman-Aiden et al. (2009), Chathoth and Zabet (2019), and Rowley et al. (2019). More specifically, we used Juicer (Durand et al. 2016) to compute the eigenvectors in 10-kb bins for all conditions (Chathoth and Zabet 2019). The sign of the correlation between the GC content and eigenvectors was used to flip the sign of the eigenvector (Imakaev et al. 2012). Bins with negative eigenvalues were assigned as a B compartment, whereas bins with positive eigenvalues were assigned as an A compartment. Chromosomes 4 and Y are relatively small, making the compartments call difficult, and thus we excluded them from the compartment analysis.

### Saddle plot and compartmentalization strength

We use the procedure similar to Naumova et al. (2013). We rank each genomic region by their eigenvector value over 30 percentile bins. Note that we only included regions that fall in the [2.5%, 97.5%] quantile interval to eliminate the effect of outliers. We then calculate the mean value over intra-arm Pearson's correlation values between regions with different percentiles. To make matrices comparable and generate saddle plots, we normalized averaged matrices using the absolute maximum values over WT and all three knockdowns. The compartment strength is calculated as the ratio of homotypic A-A and B-B interactions to heterotypic A-B and B-A interactions (Abramo et al. 2019). The ratio is calculated using the averaged signals over corner submatrices of 10 × 10 bins. Note that the compartment strength ratio uses nonnormalized signal.

### Definition of housekeeping genes

We identified 113 strong TAD borders that are conserved between Kc167 WT (Chathoth and Zabet 2019), BG3 WT, BG3 *BEAF-32*^RNAi^, BG3 *Cp190*^RNAi^ *Chro*^RNAi^, and 186 genes that are within 5 kb of these borders. We then identified expression levels for 181 of them in 85 samples (tissues, cells, conditions, or developmental stages) (Brown et al. 2014) and classified genes as housekeeping if their expression was in the top 40th percentile in all 85 samples (Corrales et al. 2017).

### RNA extraction and sequencing

RNA extraction was carried out using TRIzol according to the manufacturer's instructions. RNA was further DNase-treated and purified using an RNeasy Mini kit (Qiagen) following the manufacturer's protocol. RNA was assessed qualitatively and quantitatively using Quibit and Bioanalyzer 2100 (Agilent). Poly(A) RNA selection, library preparation, and sequencing were carried out by Novogene.

### RNA-seq analysis

Reads were first trimmed using Trimmomatic (v0.39) (Bolger et al. 2014) and then aligned to the *Drosophila melanogaster* (dm6) genome (Adams et al. 2000; dos Santos et al. 2015) using TopHat2 (v2.1.2) (Kim et al. 2013) with Bowtie 2 (v2.3.4.1) (Supplemental Table S3; Langmead and Salzberg 2012). Finally, we used Picard tools (http://broadinstitute.github.io/picard/) to deduplicate reads, HTseq (Anders et al. 2015) to count reads, and then DESeq2 (Love et al. 2014) to detect differential expressed genes. For DESeq2 we selected transcripts with at least 10 reads and used a *P*-value threshold of 0.05 and a log_2_FC threshold of 2.0 (for compartments and loops, we reduced the log_2_FC threshold to 1.0). A previous work used Affymetrix GeneChip expression analysis to quantify changes in transcription upon *BEAF-32* knockdown in BG3 cells, and they observed negligible changes in gene expression (Schwartz et al. 2012). Using RNA-seq, we found a larger number of genes displaying differential expression, but this can be explained by the increased sensitivity of RNA-seq.

### Analysis of differentially and nondifferentially expressed genes

We removed all genes that were not expressed in WT or any of the knockdowns, and then we split the genome on short regions belonging to single WT TADs or knockdown TADs. Each region was classified as follows: (1) conserved two borders if both borders of the WT TAD that contains this region are conserved; (2) conserved one border if only one of the borders moved >2 kb compared to WT position; (3) knockdown specific borders if both borders moved >2 kb compared to their WT position; and (4) fuzzy borders if both borders moved <2 kb compared to their WT position. We then performed a permutation test using the regioneR package with 1000 permutations (Gel et al. 2016).

### Pol II pausing index

We followed the method from Ramírez et al. (2018) and computed the pausing index as the ratio of the mean Pol II ChIP signal over the promoter and over the gene body. The promoter region was selected from 200 bp upstream to 50 bp downstream of the TSS and the gene body from 50 bp upstream to the gene end. Values of 0 and below were discarded.

### TF motif analysis

For BEAF-32 and Dref, we selected their corresponding motifs from MotifDb (see Supplemental Fig. S4I; https://bioconductor.org/packages/release/bioc/html/MotifDb.html). Using the ChIPanalyser Bioconductor package (Zabet and Adryan 2015; Martin and Zabet 2020), we computed the PWM sites within 2 kb of the TAD border, and, in order to include medium strength binding sites, we used a PWM threshold of 0.85.

### Occupancy heat maps

ChIP-chip data were extracted within 5 kb windows around TAD borders and winsorized (cut-off selected as the 5% quantile of negative signals and 95% quantile of positive signals), then positive signals were normalized to (0;1) and plotted. DNase-seq data were processed similarly except the negative cut-off was selected as 0 and the positive cut-off as the 75%-quantile. 3′NT-seq data was processed similarly except that negative signals were also normalized to the (-1;0) interval and plotted. All profiles were reordered with respect to the decreasing order of BEAF-32 signal summarized over a 5-kb window.

### Occupancy clustering analysis

Raw occupancy heat map signals were summarized over a 5-kb window for each TAD border. The cut-off (12.758) is defined as the 50% quantile of positive sums collected across all data sets. To separate into clusters, the following set of rules applied: (1) no signal when second and third quantiles were less than 0; (2) extra low signal when second quantile was less than 0 but third quantile was greater than 0; (3) low signal when second and third quantiles were between 0 and cut-off; (4) medium signal when second quantile was between 0 and cut-off but third quantile was greater than cut-off; (5) high signal when first quantile was less than cut-off but second quantile was greater than cut-off; and (6) extra high signal when first quantile was greater than cut-off.

### Data sets

The full list of data sets used can be found in Supplemental Tables S6–S11.

ChIP-chip: We used the ChIP-chip data sets generated and preprocessed (M values smoothed over 500 bp) by the modENCODE Consortium (Riddle et al. 2011; Schwartz et al. 2012). The fs(1)h, MED1, MED30, NippedB, vtd, SA, and SMC1 ChIP-chip data sets were downloaded from Pherson et al. (2019). To call peaks for MED1 and vtd, we first trimmed the reads using Trimmomatic (Bolger et al. 2014) (0.38), merged the two replicates (38.4 M and 16.7 M reads, respectively), aligned them to the genome using Bowtie 2 (Langmead and Salzberg 2012) (using default parameters and achieving >94% alignment rate), and then used MACS2 (Zhang et al. 2008) for peak calling (Q-value of 0.05 and using the corresponding input ChIP).

In some cases, we merged several ChIP peaks data sets: BEAF-32 (NCBI Gene Expression Omnibus [GEO; https://www.ncbi.nlm.nih.gov/geo/] accesion numbers GSE32775, GSE20811, GSE32773, and GSE32774), Cp190 (GEO accesion numbers GSE32776, GSE20814, and GSE32816), and CTCF (GEO accesion numbers GSE20767, GSE32783, and GSE32782).

DNase-seq: We used preprocessed DNase-seq profiles from the modENCODE Consortium (Kharchenko et al. 2011).

3′NT-seq: We used preprocessed 3′NT-seq in BG3 cells (GEO accesion number GSE100545) from Pherson et al. (2017).

## Data access

All Hi-C and RNA-seq data sets from this study have been submitted to the NCBI Gene Expression Omnibus (GEO; http://www.ncbi.nlm.nih.gov/geo/) under accession number GSE147059. The pipeline for Hi-C data analysis and RNA-seq is available as Supplemental Code.

## Supplementary Material

Supplemental Material
