# Supplementary material for: The role of insulators and transcription in 3D chromatin organization of flies
Source: Genome Res. 2022 Apr;32(4):682–98. doi: 10.1101/gr.275809.121 (PMC8997359; doi:10.1101/gr.275809.121)
Supplement: Supplemental Material [file supp_32_4_682__DC1.html]

The role of insulators and transcription in 3D chromatin organization of flies — Supplemental Material 

# The role of insulators and transcription in 3D chromatin organization of flies

## Supplemental Material

- Supplemental\_Fig\_S1.pdf
- Supplemental\_Fig\_S2.pdf
- Supplemental\_Fig\_S3.pdf
- Supplemental\_Fig\_S4.pdf
- Supplemental\_Fig\_S5.pdf
- Supplemental\_Fig\_S6.pdf
- Supplemental\_Fig\_S7.pdf
- Supplemental\_Fig\_S8.pdf
- Supplemental\_Fig\_S9.pdf
- Supplemental\_Fig\_S10.pdf
- Supplemental\_Fig\_S11.pdf
- Supplemental\_Fig\_S12.pdf
- Supplemental\_Fig\_S13.pdf
- Supplemental\_Fig\_S14.pdf
- Supplemental\_Fig\_S15.pdf
- Supplemental\_Fig\_S16.pdf
- Supplemental\_Table\_S1.docx
- Supplemental\_Table\_S2.docx
- Supplemental\_Table\_S3.docx
- Supplemental\_Table\_S4.docx
- Supplemental\_Table\_S5.docx
- Supplemental\_Table\_S6.docx
- Supplemental\_Table\_S7.docx
- Supplemental\_Table\_S8.docx
- Supplemental\_Table\_S9.docx
- Supplemental\_Table\_S10.docx
- Supplemental\_Table\_S11.docx
- Supplemental\_code.zip
