# Supplementary material for: The role of insulators and transcription in 3D chromatin organization of flies
Source: Genome Res. 2022 Apr;32(4):682–98. doi: 10.1101/gr.275809.121 (PMC8997359; doi:10.1101/gr.275809.121)
Supplement: Supplemental Material [file supp_gr.275809.121_Supplemental_Fig_S1.pdf]

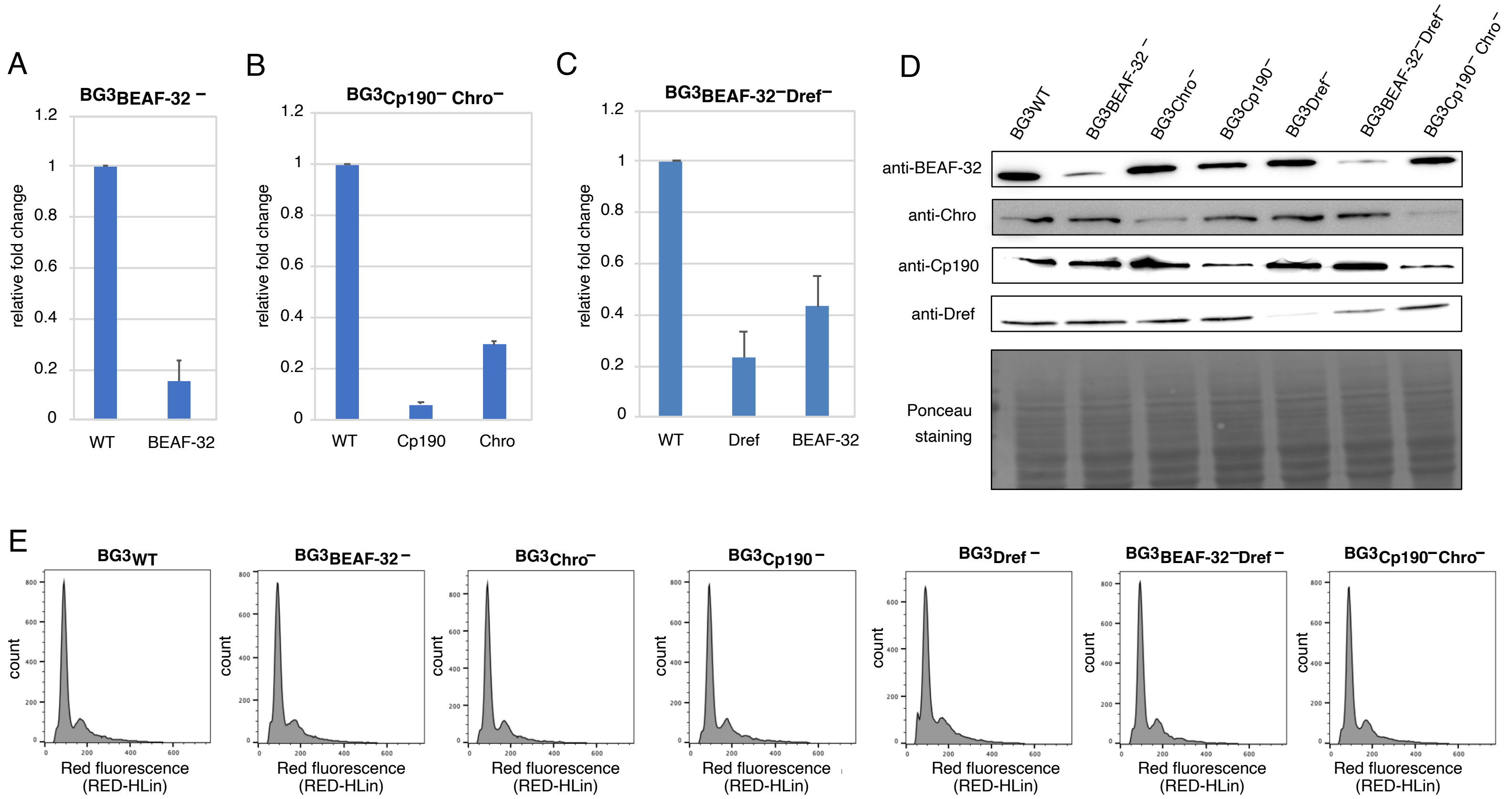
A
BG3BEAF-32 −
relative fold change
WT
BEAF-32
B
BG3Cp190− Chro−
relative fold change
WT
Cp190
Chro
C
BG3BEAF-32−Dref−
relative fold change
WT
Dref
BEAF-32
D
BG3WT
BG3BEAF-32 −
BG3Chro −
BG3Cp190−
BG3Dref−
BG3BEAF-32−Dref−
BG3Cp190− Chro −
anti-BEAF-32
anti-Chro
anti-Cp190
anti-Dref
Ponceau staining
E
BG3WT
BG3BEAF-32 −
BG3Chro−
BG3Cp190 −
BG3Dref −
BG3BEAF-32−Dref −
BG3Cp190−Chro−
count
Red fluorescence (RED-HLin)

Figure S1. *RNAi efficiency and cell cycle analysis in the three knockdowns.* RT-qPCR in (A) BEAF-32 single knockdown, (B) Cp190 Chro double knockdown and (C). BEAF-32 Dref double knockdown. Data is normalised with Rpl32 and plotted relative to WT levels. (D) Western Blots showing the depletion of BEAF-32, Chro, Cp190 and Dref following RNAi treatment in WT and BEAF-32, Cp190, Chro and Dref single and double knockdowns as indicated in the panel. (E) FACS analysis showing no effect on cell growth or cell cycle arrest in WT or BEAF-32, Cp190, Chro and Dref single and double knockdowns.
