# Supplementary material for: The role of insulators and transcription in 3D chromatin organization of flies
Source: Genome Res. 2022 Apr;32(4):682–98. doi: 10.1101/gr.275809.121 (PMC8997359; doi:10.1101/gr.275809.121)
Supplement: Supplemental Material [file supp_gr.275809.121_Supplemental_Fig_S2.pdf]

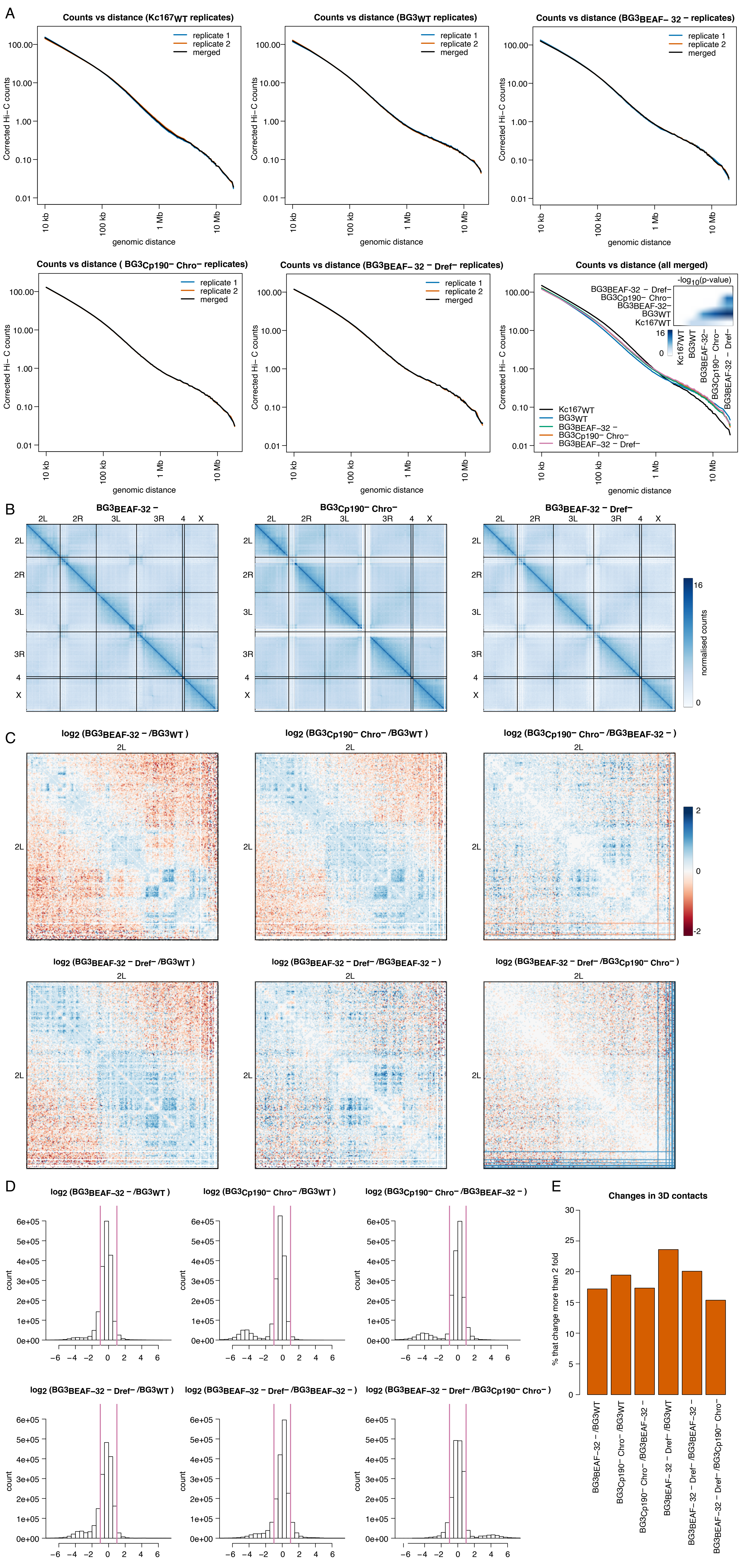
A
Counts vs distance (Kc167WT replicates)
Counts vs distance (BG3WT replicates)
Counts vs distance (BG3BEAF-32 − replicates)
Counts vs distance (BG3Cp190− Chro− replicates)
Counts vs distance (BG3BEAF-32 − Dref− replicates)
Counts vs distance (all merged)
replicate 1
replicate 2
merged
Corrected Hi-C counts
genomic distance
10 kb
100 kb
1 Mb
10 Mb
-log10(p-value)
Kc167WT
BG3WT
BG3BEAF-32 −
BG3Cp190− Chro−
BG3BEAF-32 − Dref−
B
BG3BEAF-32 −
BG3Cp190− Chro−
BG3BEAF-32 − Dref−
2L
2R
3L
3R
4
X
normalised counts
C
log2 (BG3BEAF-32 − /BG3WT)
log2 (BG3Cp190− Chro− /BG3WT)
log2 (BG3Cp190− Chro− /BG3BEAF-32 −)
log2 (BG3BEAF-32 − Dref− /BG3WT)
log2 (BG3BEAF-32 − Dref− /BG3BEAF-32 −)
log2 (BG3BEAF-32 − Dref− /BG3Cp190− Chro−)
D
count
E
Changes in 3D contacts
% that change more than 2 fold
BG3BEAF-32 − /BG3WT
BG3Cp190− Chro− /BG3WT
BG3Cp190− Chro− /BG3BEAF-32 −
BG3BEAF-32 − Dref− /BG3WT
BG3BEAF-32 − Dref− /BG3BEAF-32 −
BG3BEAF-32 − Dref− /BG3Cp190− Chro−

Figure S2. *General characterisation of Hi-C datasets.* (A) Contacts vs distance plots for HiC dataset. For each Hi-C dataset we generated two replicates and in the first five panels we compare the two replicates with a merged dataset of the two replicates for: Kc167 WT cells, BG3 WT cells, BG3 cells with BEAF-32 single knockdown, BG3 cells with Cp190 Chro double knockdown and BG3 cells with BEAF-32 Dref double knockdown. The plots confirm that there are negligible differences between the two replicates and the merged dataset in each condition. In the last panel, we plot the merge datasets for all five conditions. The plot shows that the three knockdowns in BG3 display different behaviours to WT. The inset plot shows the p-value of the paired Mann Whitney U Test and shows that all differences between the WT and knockdowns are statistically significant. (B) Genome-wide contact map of the three knockdowns (BG3 cells with BEAF-32 single knockdown, BG3 cells with Cp190 Chro double knockdown and BG3 cells with BEAF-32 Dref double knockdown) at 100 kb resolution. Each element in the matrix represents the $\log_2$ of the number of contacts between the two corresponding bins. (C) $\log_2$ ratio between the normalised number of contacts on chromosome 2L in: *(i)* BEAF-32 knockdown and WT cells, *(ii)* Cp190 Chro double knockdown and WT cells, *(iii)* Cp190 Chro double knockdown and BEAF-32 knockdown cells, *(iv)* BEAF-32 Dref double knockdown and WT cells, *(v)* BEAF-32 Dref double knockdown and BEAF-32 knockdown cells and *(vi)* BEAF-32 Dref double knockdown and Cp190 Chro double knockdown cells. Red colours indicate less contacts in the first condition and blue colours more contacts in the first condition. (D) Histograms of the $\log_2$ ratio between the normalised number of contacts genome wide in the six cases in panel (C). The vertical lines mark -1 and 1 indicating a two-fold difference between the two conditions. (E) Percentages of contacts at 100 kb bins that show at least two-fold difference between the two conditions. We also performed a Fisher's exact text and confirm that the differences in these percentages are statistically different (p-value < 0.001 in all cases).
