# Supplementary material for: The role of insulators and transcription in 3D chromatin organization of flies
Source: Genome Res. 2022 Apr;32(4):682–98. doi: 10.1101/gr.275809.121 (PMC8997359; doi:10.1101/gr.275809.121)
Supplement: Supplemental Material [file supp_gr.275809.121_Supplemental_Fig_S3.pdf]

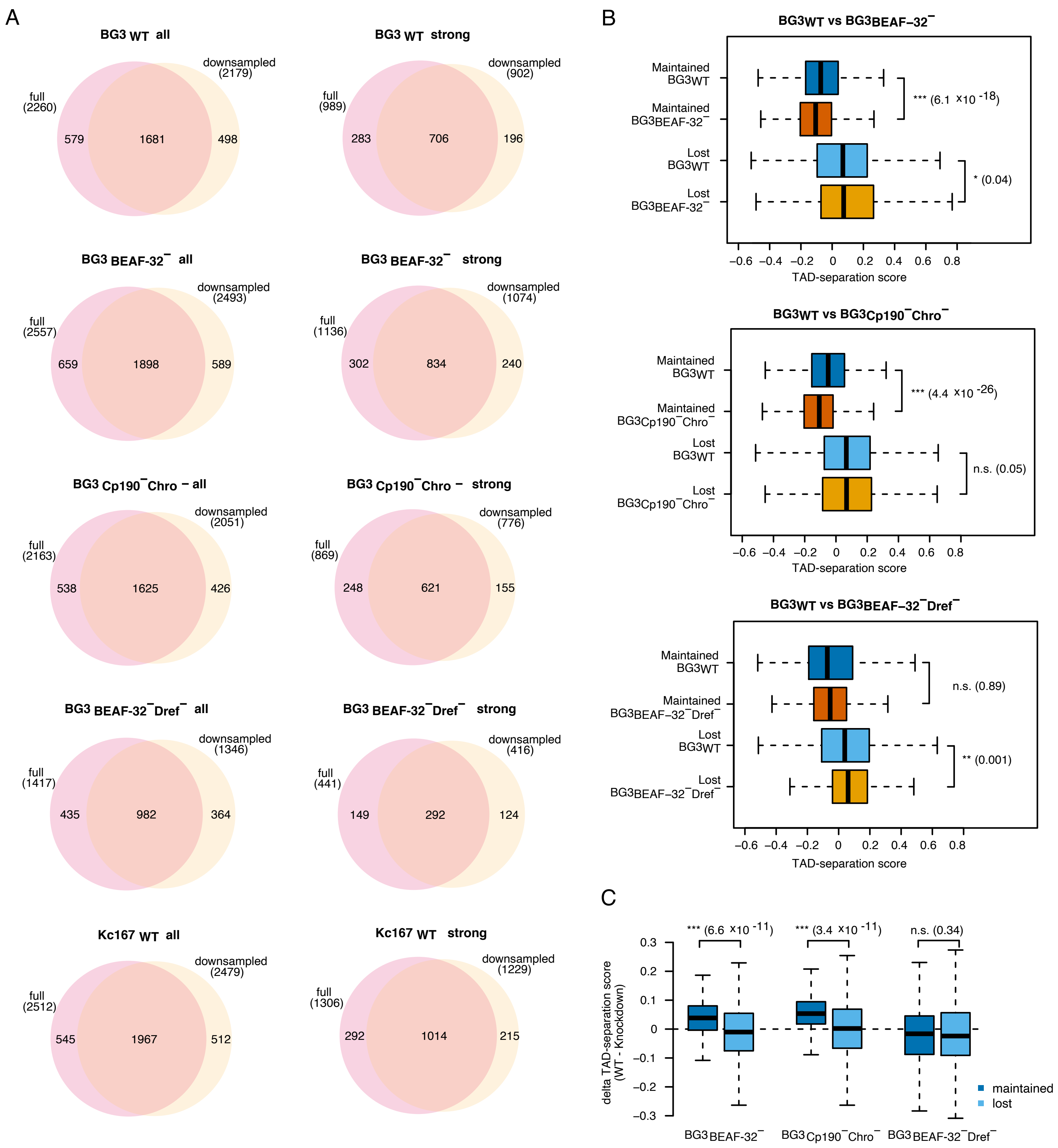
A
BG3 WT all
full (2260)
downsampled (2179)
579
1681
498
BG3 WT strong
full (989)
downsampled (902)
283
706
196
BG3 BEAF-32⁻ all
full (2557)
downsampled (2493)
659
1898
589
BG3 BEAF-32⁻ strong
full (1136)
downsampled (1074)
302
834
240
BG3 Cp190⁻Chro − all
full (2163)
downsampled (2051)
538
1625
426
BG3 Cp190⁻Chro − strong
full (869)
downsampled (776)
248
621
155
BG3 BEAF-32⁻Dref⁻ all
full (1417)
downsampled (1346)
435
982
364
BG3 BEAF-32⁻Dref⁻ strong
full (441)
downsampled (416)
149
292
124
Kc167 WT all
full (2512)
downsampled (2479)
545
1967
512
Kc167 WT strong
full (1306)
downsampled (1229)
292
1014
215
B
BG3WT vs BG3BEAF−32⁻
Maintained BG3WT
Maintained BG3BEAF-32⁻
Lost BG3WT
Lost BG3BEAF-32⁻
*** (6.1 x10 -18)
* (0.04)
TAD-separation score
BG3WT vs BG3Cp190⁻Chro⁻
Maintained BG3WT
Maintained BG3Cp190⁻Chro⁻
Lost BG3WT
Lost BG3Cp190⁻Chro⁻
*** (4.4 x10 -26)
n.s. (0.05)
TAD-separation score
BG3WT vs BG3BEAF−32⁻Dref⁻
Maintained BG3WT
Maintained BG3BEAF−32⁻Dref⁻
Lost BG3WT
Lost BG3BEAF−32⁻Dref⁻
n.s. (0.89)
** (0.001)
TAD-separation score
C
*** (6.6 x10 -11)
*** (3.4 x10 -11)
n.s. (0.34)
delta TAD-separation score (WT - Knockdown)
maintained
lost
BG3BEAF-32⁻
BG3Cp190⁻Chro⁻
BG3BEAF-32⁻Dref⁻

Figure S3. *Robustness of TAD borders.* (A) We considered both the full dataset and a downsampled dataset where we randomly removed 20% of the reads. We consider the case of all (left) and strong (right) borders separately for Hi-C datasets in BG3 WT and the three knockdowns: BEAF-32 single knockdown, Chro and Cp190 double knockdown and BEAF-32 and Dref double knockdown. We also consider the case of Kc167 WT cells. (B) Comparison of the insulation score at maintained and lost TAD borders between WT and the three knockdowns. Higher values represent less insulation in the TAD. We performed the one-sided paired Mann-Whitney *U* test. (C) Delta score (difference between WT and knockdown) at maintained and lost TAD borders in the three knockdowns. We performed the one-sided Mann-Whitney *U* test. We denoted p-values as: n.s. ≥ 0.05, * p-value < 0.05, ** < 0.01 and *** < 0.001.
