# Supplementary material for: The role of insulators and transcription in 3D chromatin organization of flies
Source: Genome Res. 2022 Apr;32(4):682–98. doi: 10.1101/gr.275809.121 (PMC8997359; doi:10.1101/gr.275809.121)
Supplement: Supplemental Material [file supp_gr.275809.121_Supplemental_Fig_S4.pdf]

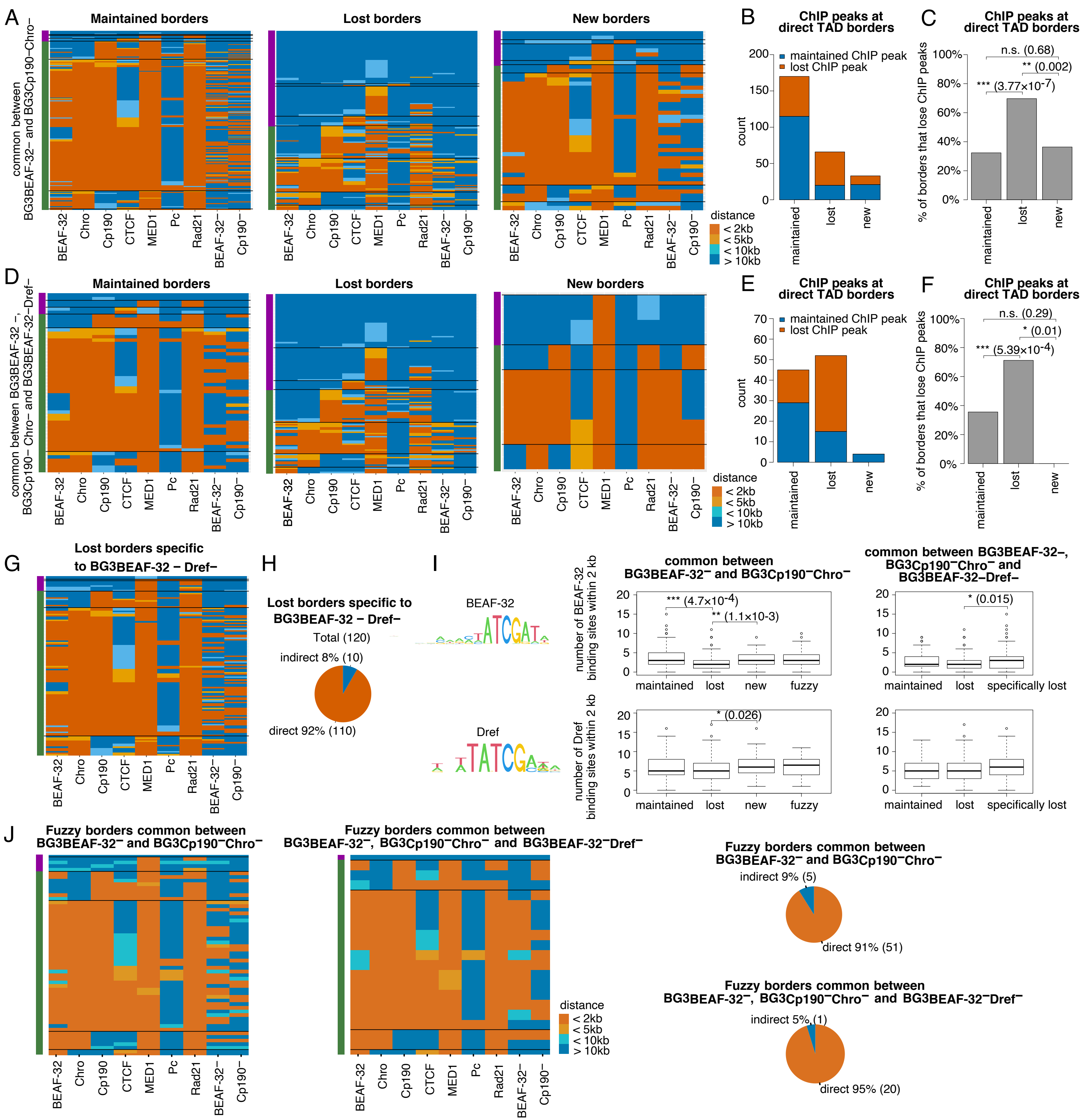

A
Maintained borders
Lost borders
New borders
common between BG3BEAF-32– and BG3Cp190–Chro–
BEAF-32
Chro
Cp190
CTCF
MED1
Pc
Rad21
BEAF-32–
Cp190–
distance
< 2kb
< 5kb
< 10kb
> 10kb
B
ChIP peaks at direct TAD borders
maintained ChIP peak
lost ChIP peak
count
maintained
lost
new
C
ChIP peaks at direct TAD borders
% of borders that lose ChIP peaks
n.s. (0.68)
** (0.002)
*** (3.77x10-7)
D
common between BG3BEAF-32 –, BG3Cp190– Chro– and BG3BEAF-32–Dref–
Maintained borders
Lost borders
New borders
E
ChIP peaks at direct TAD borders
F
ChIP peaks at direct TAD borders
n.s. (0.29)
* (0.01)
*** (5.39x10-4)
G
Lost borders specific to BG3BEAF-32 – Dref–
H
Lost borders specific to BG3BEAF-32 – Dref–
Total (120)
indirect 8% (10)
direct 92% (110)
I
BEAF-32
Dref
number of BEAF-32 binding sites within 2 kb
number of Dref binding sites within 2 kb
common between BG3BEAF-32– and BG3Cp190–Chro–
*** (4.7x10-4)
** (1.1x10-3)
* (0.026)
maintained
lost
new
fuzzy
common between BG3BEAF-32–, BG3Cp190–Chro– and BG3BEAF-32–Dref–
* (0.015)
specifically lost
J
Fuzzy borders common between BG3BEAF-32– and BG3Cp190–Chro–
Fuzzy borders common between BG3BEAF-32–, BG3Cp190–Chro– and BG3BEAF-32–Dref–
indirect 9% (5)
direct 91% (51)
indirect 5% (1)
direct 95% (20)

Figure S4. *Direct and indirect TAD borders.* We considered separately the cases of maintained, lost and new borders that are: (A-C) common between BEAF-32 single knockdown and Cp190 and Chro double knockdown and (D-F) common between BEAF-32 single knockdown, Cp190 and Chro double knockdown and BEAF-32 and Dref double knockdown. (A and D) Heatmaps plotting the distance of the closest ChIP peak from a maintained, lost and new border for: BEAF-32 (WT and BEAF-32 knockdown), Chro (WT), Cp190 (WT and Cp190 knockdown), CTCF (WT), MED1 (WT), Pc (WT) and Rad21 (WT). Green bar on the side of each heatmap marks the direct borders (borders that show binding of BEAF-32, Chro and/or Cp190 in WT cells), while purple indirect borders (all other borders). (B and E) number of TAD borders that have BEAF-32 or Cp190 ChIP in WT cells and lose or maintain those peaks in BEAF-32 and Cp190 single knockdowns. (C and F) percentage of TAD borders that have BEAF-32 or Cp190 ChIP in WT and lose them in the in BEAF-32 and Cp190 single knockdowns. We performed a Fisher's exact test and the corresponding p-value is displayed inside the plots. (G) Heatmap plotting the distance of the closest ChIP peak from lost TAD borders that are specific to BEAF-32 and Dref double knockdown for same proteins as in (A and D). Green bar on the side of each heatmap marks the direct borders (borders that show binding of BEAF-32, Chro and/or Cp190 in WT cells), while purple indirect borders (all other borders). (H) Total number and proportions of TAD borders that are specifically lost in BEAF-32 and Dref double knockdown and have binding of BEAF-32, Cp190 and/or Chro. (I) We counted the number of BEAF-32 and Dref binding sites within 2 kb of the TAD borders (see Materials and Methods). We considered the case of TAD borders: (left panels) common between BEAF-32 single knockdown and Cp190 Chro double knockdown and (right panels) common between BEAF-32 single knockdown, Cp190 Chro double knockdown and BEAF-32 Dref double knockdown. Note that for the latter, we also considered the case of TAD borders that are specifically lost in BEAF-32 Dref double knockdown. We performed a Mann-Whitney *U* test (p-value: n.s. ≥ 0.05, * p-value < 0.05, ** < 0.01 and *** < 0.001) and, for clarity, we included on the plot only the values for the statistically significant differences (p-value < 0.05). (J) Heatmap plotting the distance of the closest ChIP peak from fuzzy TAD borders using the same proteins as in (A and D). We consider separately the case of: *(i)* fuzzy borders common between BEAF-32 single knockdown and Cp190 and Chro double knockdown and *(ii)* fuzzy borders common between BEAF-32 single knockdown, Cp190 and Chro double knockdown and BEAF-32 and Dref double knockdown. Pie charts represent the number and percentage of direct and indirect borders.
