# Supplementary material for: The role of insulators and transcription in 3D chromatin organization of flies
Source: Genome Res. 2022 Apr;32(4):682–98. doi: 10.1101/gr.275809.121 (PMC8997359; doi:10.1101/gr.275809.121)
Supplement: Supplemental Material [file supp_gr.275809.121_Supplemental_Fig_S5.pdf]

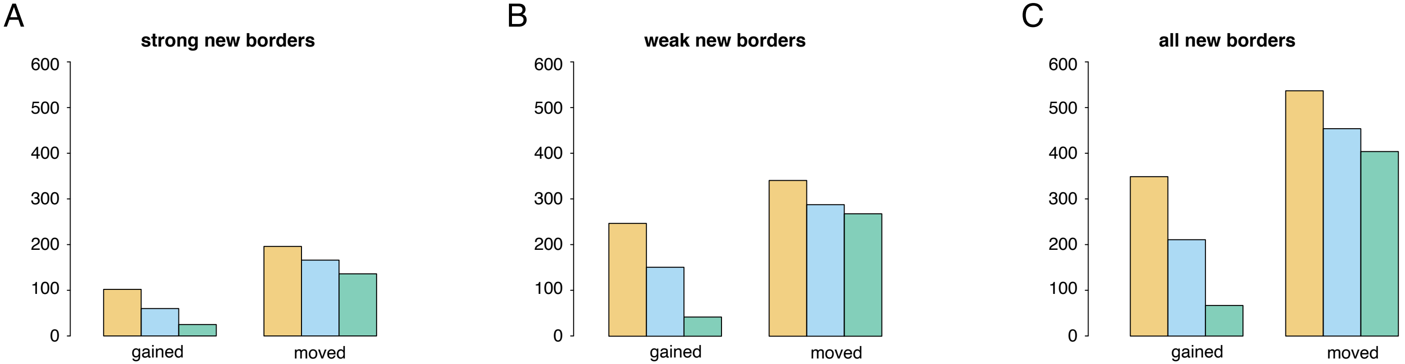

	gained	moved
BG3BEAF−32 −	102	196
BG3Cp190−Chro−	60	166
BG3BEAF−32 −Dref−	25	136

	gained	moved
BG3BEAF−32 −	247	341
BG3Cp190−Chro−	151	288
BG3BEAF−32 −Dref−	42	268

	gained	moved
BG3BEAF−32 −	349	537
BG3Cp190−Chro−	211	454
BG3BEAF−32 −Dref−	67	404

Figure S5. *Classification of new TAD borders in the knockdowns.* New borders in the three knockdowns (BEAF-32 knockdown, Cp190 Chro double knockdown and BEAF-32 Dref double knockdown) can be gained as a consequence appearing inside a WT TAD (splitting a TAD) or moved when they correspond to relocation of a WT TAD border. We considered the cases of: (A) new strong borders; (B) new weak borders; (C) all new borders.
