# Supplementary material for: The role of insulators and transcription in 3D chromatin organization of flies
Source: Genome Res. 2022 Apr;32(4):682–98. doi: 10.1101/gr.275809.121 (PMC8997359; doi:10.1101/gr.275809.121)
Supplement: Supplemental Material [file supp_gr.275809.121_Supplemental_Fig_S6.pdf]

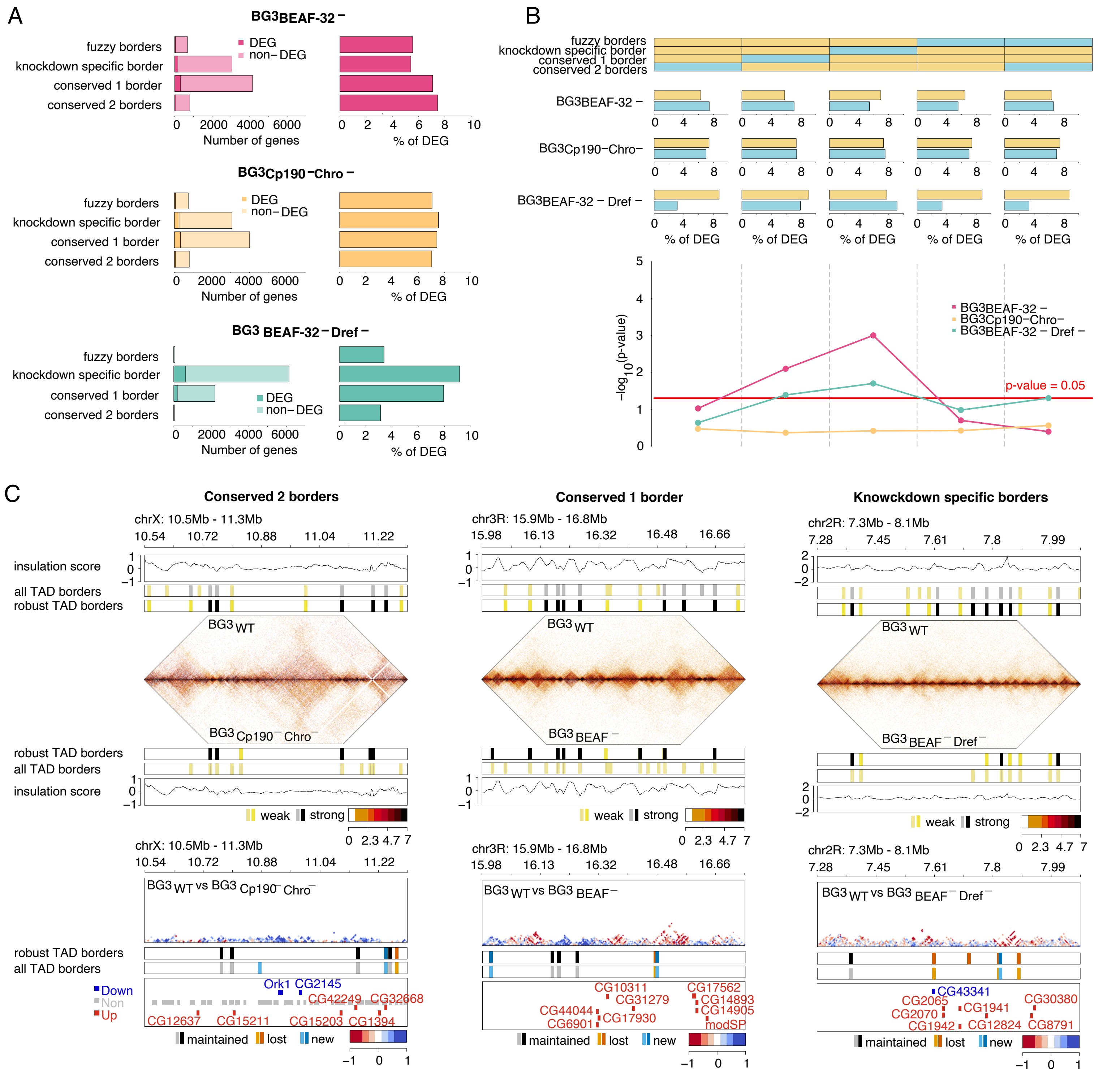

A
BG3BEAF-32 −
fuzzy borders
knockdown specific border
conserved 1 border
conserved 2 borders
DEG
non− DEG
Number of genes
% of DEG
BG3Cp190−Chro −
BG3 BEAF-32 − Dref −
B
BG3BEAF-32 −
BG3Cp190−Chro−
BG3BEAF-32 − Dref −
−log10(p-value)
p-value = 0.05
C
Conserved 2 borders
Conserved 1 border
Knowckdown specific borders
chrX: 10.5Mb - 11.3Mb
chr3R: 15.9Mb - 16.8Mb
chr2R: 7.3Mb - 8.1Mb
insulation score
all TAD borders
robust TAD borders
BG3 WT
BG3 Cp190− Chro−
BG3 BEAF −
BG3 BEAF − Dref −
weak
strong
BG3 WT vs BG3 Cp190− Chro−
BG3 WT vs BG3 BEAF−
BG3 WT vs BG3 BEAF − Dref −
Down
Non
Up
Ork1
CG2145
CG42249
CG32668
CG12637
CG15211
CG15203
CG1394
CG10311
CG31279
CG17562
CG14893
CG14905
CG44044
CG6901
CG17930
modSP
CG43341
CG2065
CG2070
CG1942
CG1941
CG12824
CG30380
CG8791
maintained
lost
new

Figure S6. *Comparison of differentially and non-differentially expressed genes within different TAD classes in the knockdowns.* (A) Count and proportion of DEG and non-DEG in the three knockdowns (BEAF-32 knockdown, Cp190 Chro double knockdown and BEAF-32 Dref double knockdown) within TADs that: are fully conserved; lose one or both borders; have slightly shifted borders in the knockdowns. (B) We group different TAD classes in two subgroups (blue and yellow) and performed a permutation test to investigate if DEG overlap with any of the class more than expected by chance and plot the corresponding proportion of DEG over non-DEG for each group together with the corresponding -$\log_{10}$ p-values. (C) Examples of DEGs inside TADs that have: *(i)* both borders conserved, *(ii)* one border conserved or *(iii)* both borders are knockdown specific. From top to bottom we plot the insulation score, TAD borders in full dataset (grey are strong and yellow are weak), TAD borders recovered both in full and downsampled dataset (black are strong and yellow are weak) and contact map in WT cells. We also plot a mirror plot in the knockdowns and $\log_2$ fold change between WT and knockdown. To compute the $\log_2$FC, we followed the steps and parameters recommended in diffHiC package (Lun and Smyth 2015). Briefly, we consider individual replicates and use edgeR package (Robinson et al. 2010) to compute the $\log_2$FC in 5 kb bins. On the last row, we plot the position of the non-differentially expressed (grey), upregulated (blue) and downregulated (red) genes.
