# Supplementary material for: The role of insulators and transcription in 3D chromatin organization of flies
Source: Genome Res. 2022 Apr;32(4):682–98. doi: 10.1101/gr.275809.121 (PMC8997359; doi:10.1101/gr.275809.121)
Supplement: Supplemental Material [file supp_gr.275809.121_Supplemental_Fig_S7.pdf]

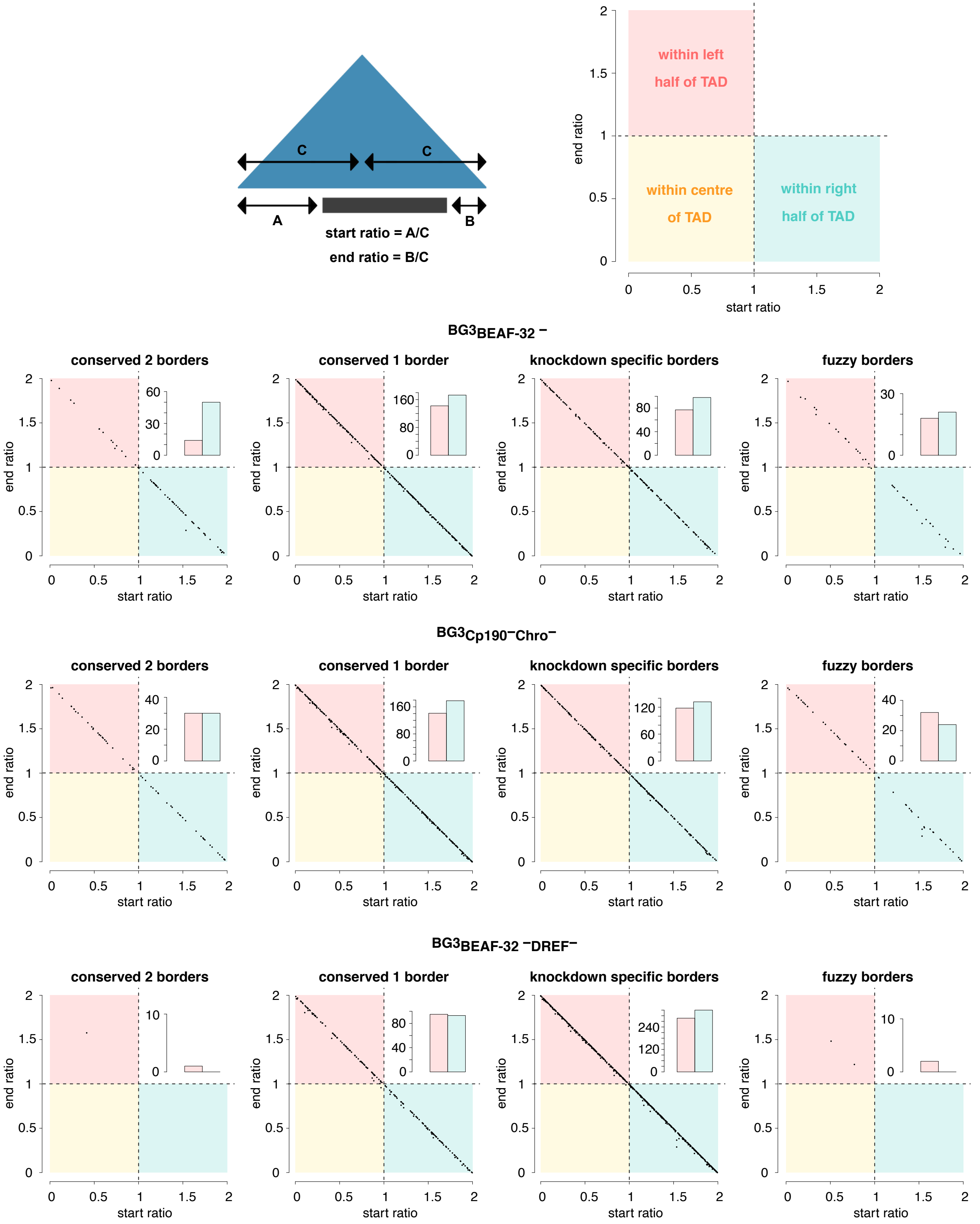

C
C
A
B
start ratio = A/C
end ratio = B/C
within left
half of TAD
within centre
of TAD
within right
half of TAD
end ratio
start ratio
BG3BEAF-32 −
BG3Cp190−Chro−
BG3BEAF-32 −DREF−
conserved 2 borders
conserved 1 border
knockdown specific borders
fuzzy borders

Figure S7. *The allocation of differentially expressed genes within robust TADs.* Allocation and count of differentially expressed genes within robust TADs in three knockdowns (BEAF-32 knockdown, Cp190 and Chro double knockdown, BEAF-32 Dref double knockdown) based on start and end ratios. The start ratio is defined as a distance from the left border of the TAD to the start position of the gene divided by the half of TAD size, where a start ratio bigger than 1 means that the gene is allocated on the right side of the TAD (green square). The end ratio is defined as a distance from the right border of the TAD to the end position of the gene divided by the half of TAD size, where an end ratio bigger than 1 indicates the gene allocated on the left side of the TAD (red square). Genes having both start and end ratio less than 1 are allocated within TAD centre (yellow square). The majority of differentially expressed genes occupy less than half of the TAD. Only couple of genes are allocated within TAD centre – they are very close to point (1,1) indicating relatively short genes. Majority of genes are allocated either on the left or the right half of the TAD with no strong bias towards TAD borders.
