# Supplementary material for: The role of insulators and transcription in 3D chromatin organization of flies
Source: Genome Res. 2022 Apr;32(4):682–98. doi: 10.1101/gr.275809.121 (PMC8997359; doi:10.1101/gr.275809.121)
Supplement: Supplemental Material [file supp_gr.275809.121_Supplemental_Fig_S8.pdf]

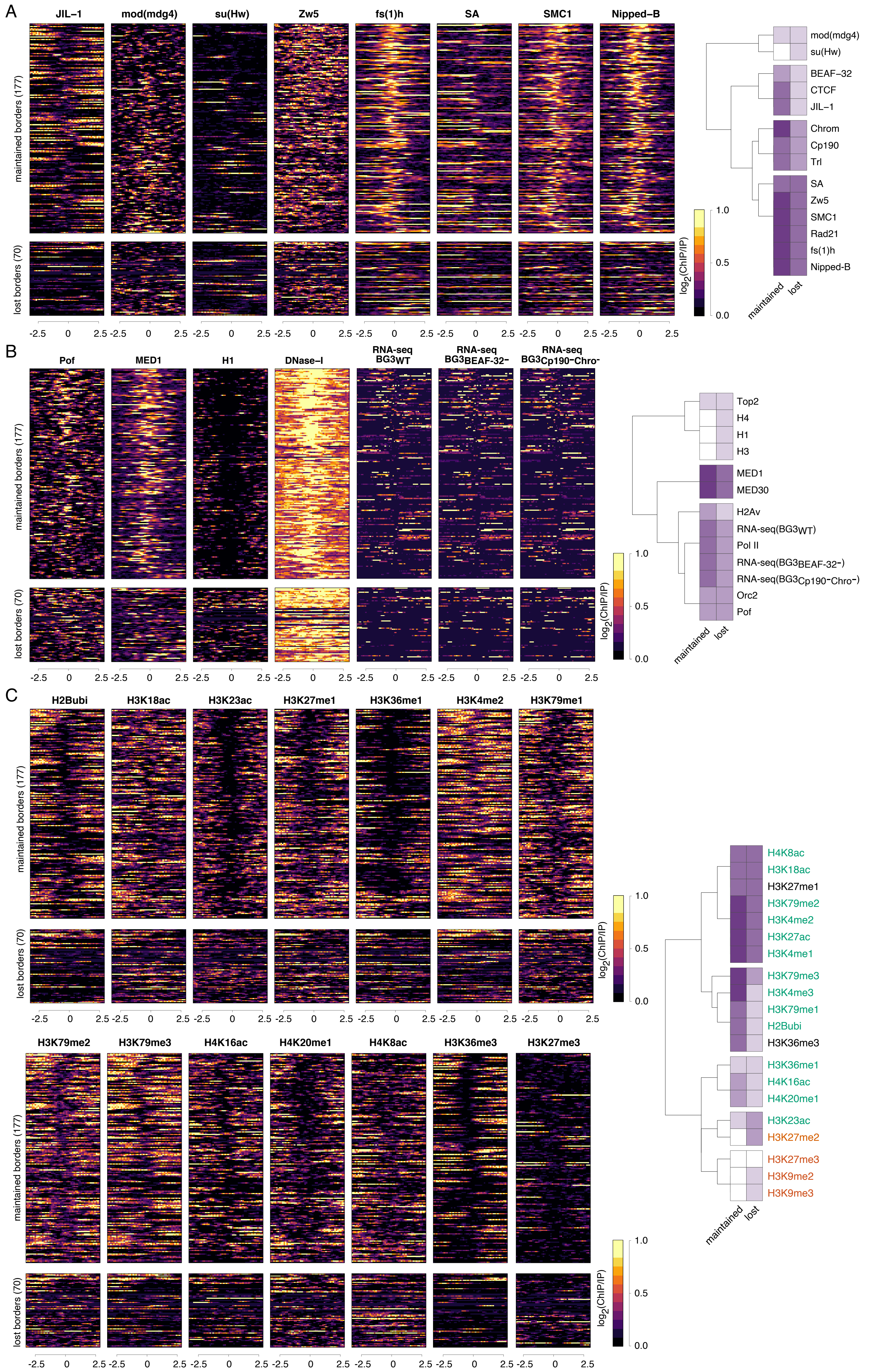

A
JIL−1
mod(mdg4)
su(Hw)
Zw5
fs(1)h
SA
SMC1
Nipped−B
maintained borders (177)
lost borders (70)
-2.5
0
2.5
log2(ChIP/IP)
1.0
0.5
0.0
mod(mdg4)
su(Hw)
BEAF−32
CTCF
JIL−1
Chrom
Cp190
Trl
SA
Zw5
SMC1
Rad21
fs(1)h
Nipped-B
maintained
lost
B
Pof
MED1
H1
DNase−I
RNA-seq BG3WT
RNA-seq BG3BEAF-32-
RNA-seq BG3Cp190-Chro-
Top2
H4
H1
H3
MED1
MED30
H2Av
RNA-seq(BG3WT)
Pol II
RNA-seq(BG3BEAF-32-)
RNA-seq(BG3Cp190-Chro-)
Orc2
Pof
C
H2Bubi
H3K18ac
H3K23ac
H3K27me1
H3K36me1
H3K4me2
H3K79me1
H3K79me2
H3K79me3
H4K16ac
H4K20me1
H4K8ac
H3K36me3
H3K27me3
H4K8ac
H3K18ac
H3K27me1
H3K79me2
H3K4me2
H3K27ac
H3K4me1
H3K79me3
H3K4me3
H3K79me1
H2Bubi
H3K36me3
H3K36me1
H4K16ac
H4K20me1
H3K23ac
H3K27me2
H3K27me3
H3K9me2
H3K9me3

Figure S8. *Additional chromatin features enrichment at TAD borders.* (A) Profiles of architectural proteins (JIL-1, mod(mdg4), su(Hw), Zw5, fs(1)h, SA, SMC1 and Nipped-B) around direct maintained and lost TAD borders. The right panel plots clustering of the signal at maintained and lost TAD borders (see Materials and Methods). (B) Profiles of accessibility (H1 and DNase-I) and transcription (MED1 and Pof) at maintained and lost TAD borders. In addition, we plot RNA-seq signal in WT, BEAF-32 knockdown and Cp190 and Chro double knockdown. (C) Profiles of histone modifications (H2Bubi, H3K18ac, H3K23ac, H3K27me1, H3K36me1, H3K4me2, H3K79me1, H3K79me2, H3K79me3, H4K16ac, H4K20me1, H4K8ac, H3K36me3 and H3K27me3) at direct maintained and lost TAD borders. In the clustering analysis, we marked with red histone modifications associated with dense chromatin and with green histone modifications associated to open chromatin. Histone modifications that have been reported to be associated with both open and dense chromatin were marked by black.
