# Supplementary material for: The role of insulators and transcription in 3D chromatin organization of flies
Source: Genome Res. 2022 Apr;32(4):682–98. doi: 10.1101/gr.275809.121 (PMC8997359; doi:10.1101/gr.275809.121)
Supplement: Supplemental Material [file supp_gr.275809.121_Supplemental_Fig_S9.pdf]

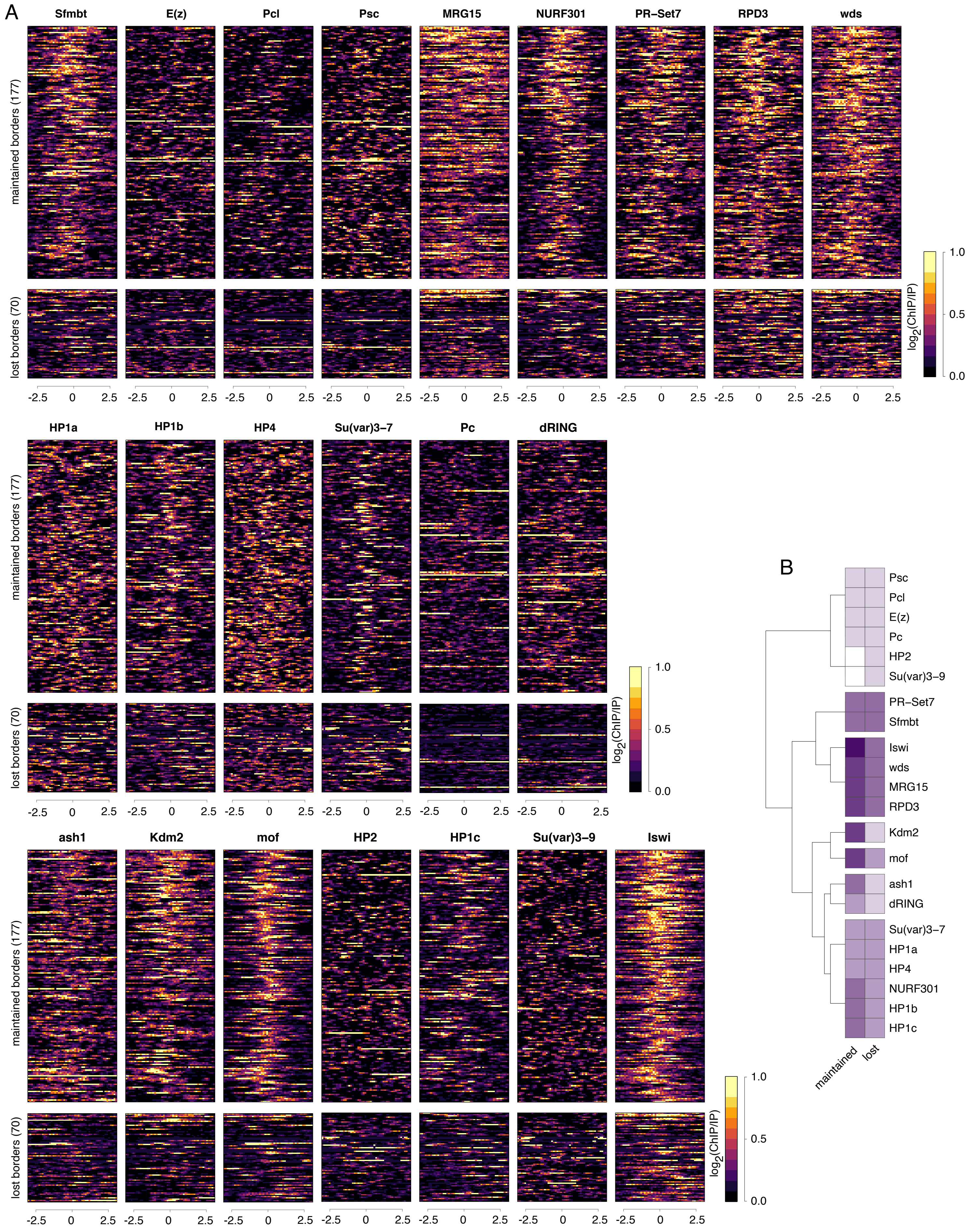
A
Sfmbt
E(z)
Pcl
Psc
MRG15
NURF301
PR-Set7
RPD3
wds
maintained borders (177)
lost borders (70)
-2.5
0
2.5
log2(ChIP/IP)
0.0
0.5
1.0
HP1a
HP1b
HP4
Su(var)3-7
Pc
dRING
ash1
Kdm2
mof
HP2
HP1c
Su(var)3-9
Iswi
B
Psc
Pcl
E(z)
Pc
HP2
Su(var)3-9
PR-Set7
Sfmbt
Iswi
wds
MRG15
RPD3
Kdm2
mof
ash1
dRING
Su(var)3-7
HP1a
HP4
NURF301
HP1b
HP1c
maintained
lost

Figure S9. *Enrichment of other proteins/complexes at TAD borders.* (A) Profiles of Pc, dRING, ash1, Kdm2, mof, HP2, HP1c, Su(var)3-9, Iswi, Sfmbt, E(z), Pcl, Psc, MRG15, NURF301, PR-Set7, RPD3, wds, HP1a, HP1b, HP4 and Su(var)3-7 at direct maintained and lost TAD borders. (B) Clustering of the signal at direct maintained and lost TAD borders.
