# Supplementary material for: The role of insulators and transcription in 3D chromatin organization of flies
Source: Genome Res. 2022 Apr;32(4):682–98. doi: 10.1101/gr.275809.121 (PMC8997359; doi:10.1101/gr.275809.121)
Supplement: Supplemental Material [file supp_gr.275809.121_Supplemental_Fig_S10.pdf]

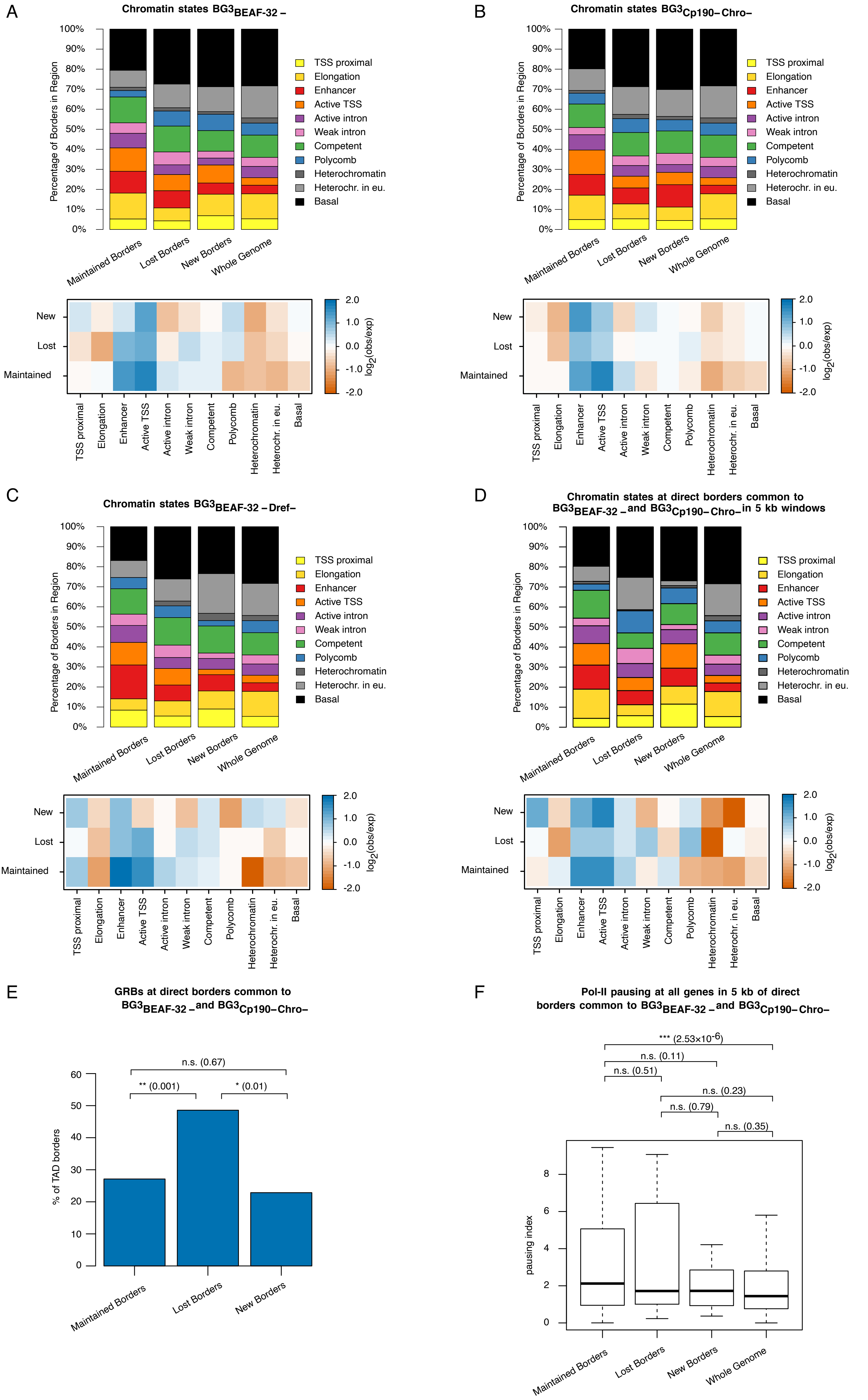
A
Chromatin states BG3BEAF-32 –
Percentage of Borders in Region
100%
90%
80%
70%
60%
50%
40%
30%
20%
10%
0%
Maintained Borders
Lost Borders
New Borders
Whole Genome
TSS proximal
Elongation
Enhancer
Active TSS
Active intron
Weak intron
Competent
Polycomb
Heterochromatin
Heterochr. in eu.
Basal
New
Lost
Maintained
2.0
1.0
0.0
-1.0
-2.0
log2(obs/exp)
B
Chromatin states BG3Cp190– Chro–
C
Chromatin states BG3BEAF-32 – Dref–
D
Chromatin states at direct borders common to BG3BEAF-32 – and BG3Cp190– Chro– in 5 kb windows
E
GRBs at direct borders common to BG3BEAF-32 – and BG3Cp190– Chro–
% of TAD borders
60
50
40
30
20
10
0
n.s. (0.67)
** (0.001)
* (0.01)
Maintained Borders
Lost Borders
New Borders
F
Pol-II pausing at all genes in 5 kb of direct borders common to BG3BEAF-32 – and BG3Cp190– Chro–
*** (2.53×10-6)
n.s. (0.11)
n.s. (0.51)
n.s. (0.23)
n.s. (0.79)
n.s. (0.35)
pausing index
8
6
4
2
0
Maintained Borders
Lost Borders
New Borders
Whole Genome

Figure S10. *Chromatin state annotation at maintained, lost and new TAD borders.* We computed the overlap between each maintained, lost and new robust TAD border (see Fig. 1B and and Fig. 2A) in the three knockdowns with chromatin states: (A) BEAF-32 single knockdown, (B) Cp190 and Chro double knockdown and (C) BEAF-32 and Dref double knockdown. Top panels: the percentage of the overlap between the different chromatin states and maintained, lost and new robust TAD borders and whole genome. Bottom panels: $\log_2$(observed/expected), where observed is the percentage in maintained, lost or new classes and expected is the whole genome distribution. (D) Same as in (A-C) for maintained, lost and new direct borders that were common between BEAF-32 single knockdown and Cp190 and Chro double knockdown. (E) Gene Regulatory Blocks within 2 kb of different categories of direct borders: maintained, lost and new borders common between BEAF-32 single knockdown and Cp190 and Chro double knockdown. We performed a Fisher's exact test (p-value: n.s. ≥ 0.05, * p-value < 0.05, ** < 0.01 and *** < 0.001). (F) Pol II pausing at maintained, lost and new direct borders that were common in BEAF-32 single knockdown and Cp190 and Chro double knockdown. We performed a Mann-Whitney *U* test (p-value: n.s. ≥ 0.05, * p-value < 0.05, ** < 0.01 and *** < 0.001).
