# Supplementary material for: The role of insulators and transcription in 3D chromatin organization of flies
Source: Genome Res. 2022 Apr;32(4):682–98. doi: 10.1101/gr.275809.121 (PMC8997359; doi:10.1101/gr.275809.121)
Supplement: Supplemental Material [file supp_gr.275809.121_Supplemental_Fig_S11.pdf]

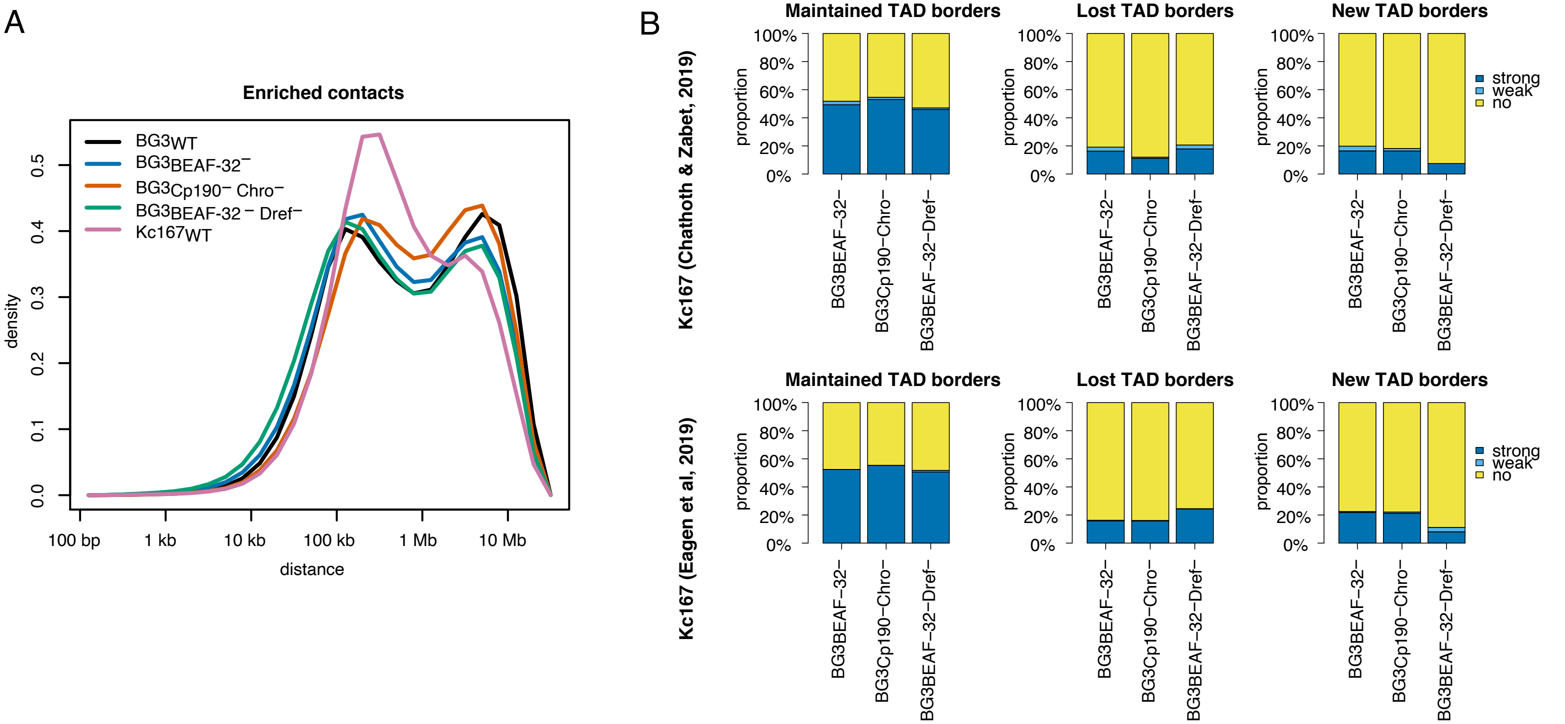

A
Enriched contacts
BG3WT
BG3BEAF-32−
BG3Cp190− Chro−
BG3BEAF-32− Dref−
Kc167WT
density
distance
0.0
0.1
0.2
0.3
0.4
0.5
100 bp
1 kb
10 kb
100 kb
1 Mb
10 Mb
B
Kc167 (Chathoth & Zabet, 2019)
Kc167 (Eagen et al, 2019)
Maintained TAD borders
Lost TAD borders
New TAD borders
proportion
0%
20%
40%
60%
80%
100%
strong
weak
no
BG3BEAF−32−
BG3Cp190−Chro−
BG3BEAF−32−Dref−

Figure S11. *Comparison of the 3D chromatin organisation in the knockdowns with 3D chromatin organisation in Kc167 cells.* (A) Density distribution of enriched number of contacts. We highlight the density plot of the enriched contacts instead of counts. This was necessary in order to take into account the differences generated by sequencing depth of the libraries for the different knockdowns. For Kc167 we used data from (Chathoth and Zabet 2019). (B) Proportion of robust TAD borders that are maintained in the three knockdowns and are also present in Kc167 cells as either strong or weak borders. Top panels: annotation of robust TAD borders in Kc167 using data from (Chathoth and Zabet 2019). Bottom panels: annotation of TAD borders in Kc167 using a very high sequencing depth library from (Eagen et al. 2017).
