# Supplementary material for: The role of insulators and transcription in 3D chromatin organization of flies
Source: Genome Res. 2022 Apr;32(4):682–98. doi: 10.1101/gr.275809.121 (PMC8997359; doi:10.1101/gr.275809.121)
Supplement: Supplemental Material [file supp_gr.275809.121_Supplemental_Fig_S12.pdf]

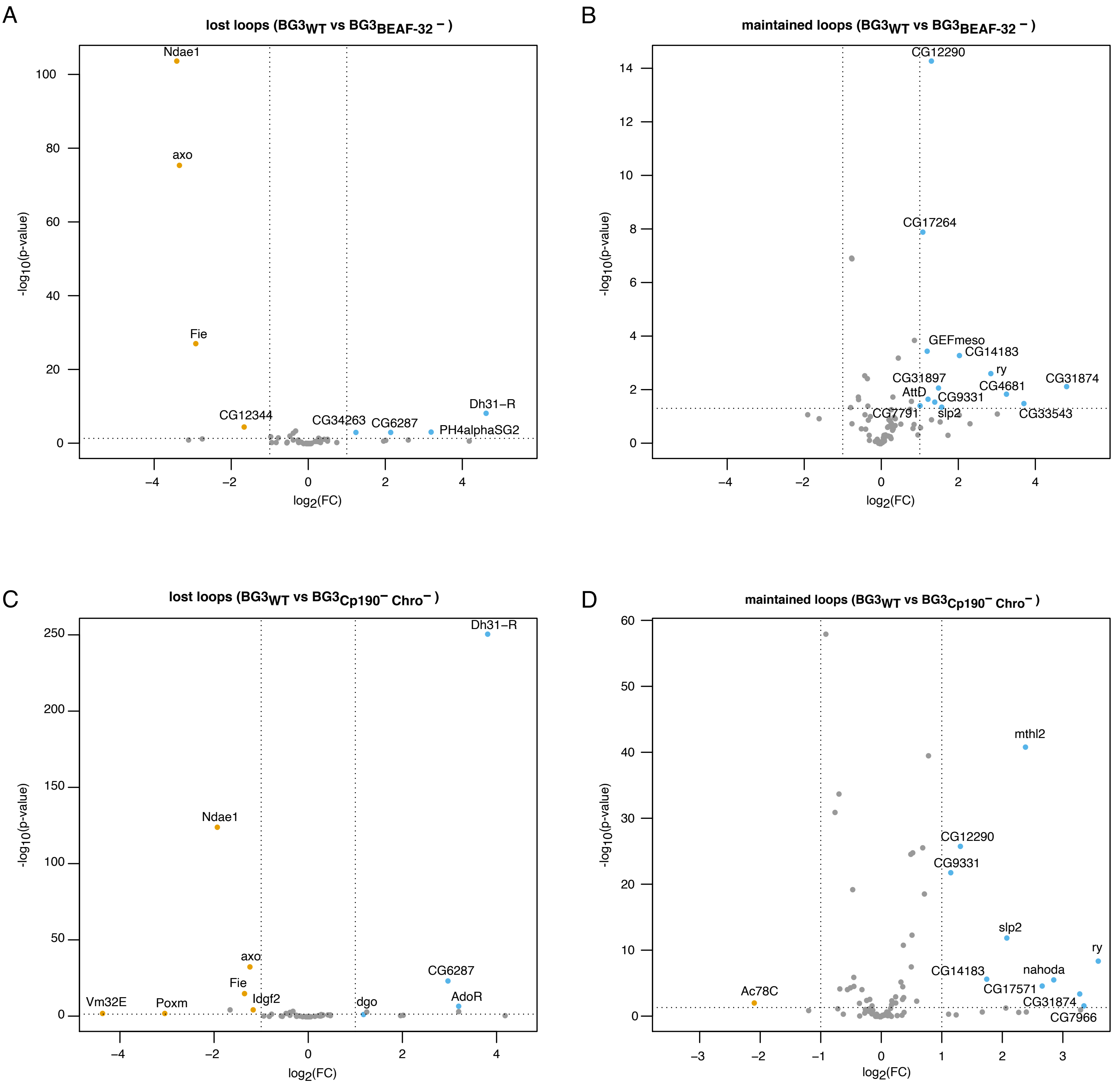
A
lost loops (BG3WT vs BG3BEAF-32 −)
Ndae1
axo
Fie
CG12344
CG34263
CG6287
Dh31−R
PH4alphaSG2
-log10(p-value)
log2(FC)
B
maintained loops (BG3WT vs BG3BEAF-32 −)
CG12290
CG17264
GEFmeso
CG14183
ry
CG31897
CG31874
CG4681
AttD
CG9331
CG7791
slp2
CG33543
-log10(p-value)
log2(FC)
C
lost loops (BG3WT vs BG3Cp190− Chro−)
Dh31−R
Ndae1
axo
Fie
Idgf2
Vm32E
Poxm
dgo
CG6287
AdoR
-log10(p-value)
log2(FC)
D
maintained loops (BG3WT vs BG3Cp190− Chro−)
mthl2
CG12290
CG9331
slp2
ry
CG14183
nahoda
CG17571
CG31874
CG7966
Ac78C
-log10(p-value)
log2(FC)

Figure S12. *Differential expression of genes located at chromatin loops.* Volcano plots of the $-\log_{10}$ of the adjusted p-value as a function of $\log_2$ fold change. We considered all genes located at lost chromatin loops (A and C) and maintained chromatin loops (B and D) in BEAF-32 single knockdown (A and B) and Cp190 and Chro double knockdown (C and D).
