# Supplementary material for: The role of insulators and transcription in 3D chromatin organization of flies
Source: Genome Res. 2022 Apr;32(4):682–98. doi: 10.1101/gr.275809.121 (PMC8997359; doi:10.1101/gr.275809.121)
Supplement: Supplemental Material [file supp_gr.275809.121_Supplemental_Fig_S13.pdf]

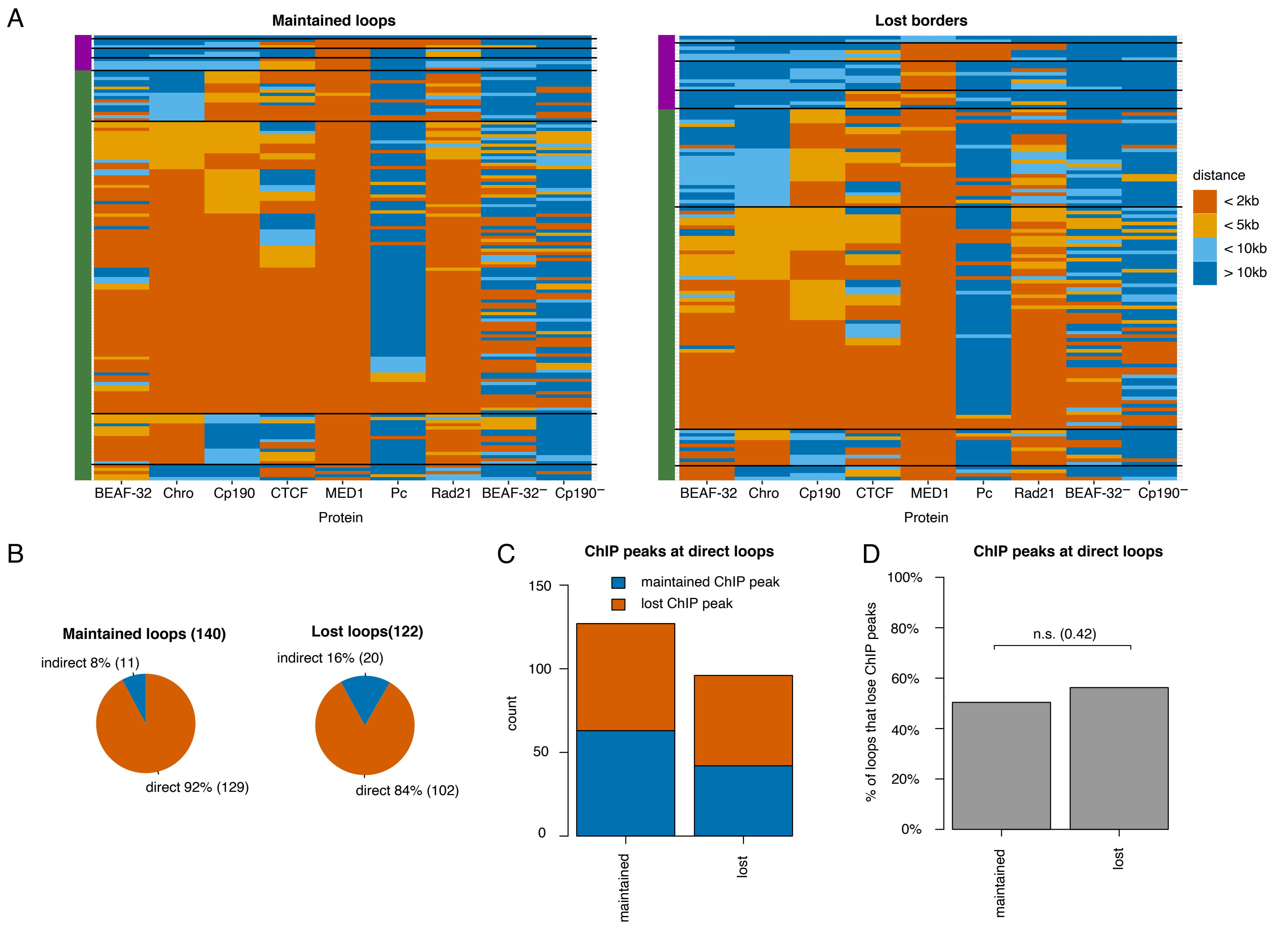
A
Maintained loops
Lost borders
distance
< 2kb
< 5kb
< 10kb
> 10kb
BEAF-32
Chro
Cp190
CTCF
MED1
Pc
Rad21
BEAF-32⁻
Cp190⁻
Protein
B
Maintained loops (140)
indirect 8% (11)
direct 92% (129)
Lost loops(122)
indirect 16% (20)
direct 84% (102)
C
ChIP peaks at direct loops
maintained ChIP peak
lost ChIP peak
count
150
100
50
0
maintained
lost
D
ChIP peaks at direct loops
% of loops that lose ChIP peaks
100%
80%
60%
40%
20%
0%
n.s. (0.42)
maintained
lost

Figure S13. *Direct and indirect chromatin loops.* We considered separately the cases of maintained and lost chromatin loops. (A) Heatmaps plotting the distance of the closest ChIP peak from a maintained or lost loop for: BEAF-32 (WT and BEAF-32 knockdown), Chro (WT), Cp190 (WT and Cp190 knockdown), CTCF (WT), MED1 (WT), Pc (WT) and Rad21 (WT). Green bar on the side of each heatmap marks direct loops (loops that show binding of BEAF-32, Chro and/or Cp190 in WT cells), while purple indirect loops (all other borders). (B) Percentage of maintained and lost chromatin loops that have direct binding of BEAF-32, Cp190 and Chro. (C) number of chromatin loops that have BEAF-32 or Cp190 ChIP in WT cells and lose those peaks in BEAF-32 and Cp190 single knockdowns. (D) Percentage of chromatin loops that have BEAF-32 or Cp190 ChIP in WT and lose them in the in BEAF-32 and Cp190 single knockdowns. We performed a Fisher's exact test and the corresponding p-value is displayed above the barplots.
