# Supplementary material for: The role of insulators and transcription in 3D chromatin organization of flies
Source: Genome Res. 2022 Apr;32(4):682–98. doi: 10.1101/gr.275809.121 (PMC8997359; doi:10.1101/gr.275809.121)
Supplement: Supplemental Material [file supp_gr.275809.121_Supplemental_Fig_S14.pdf]

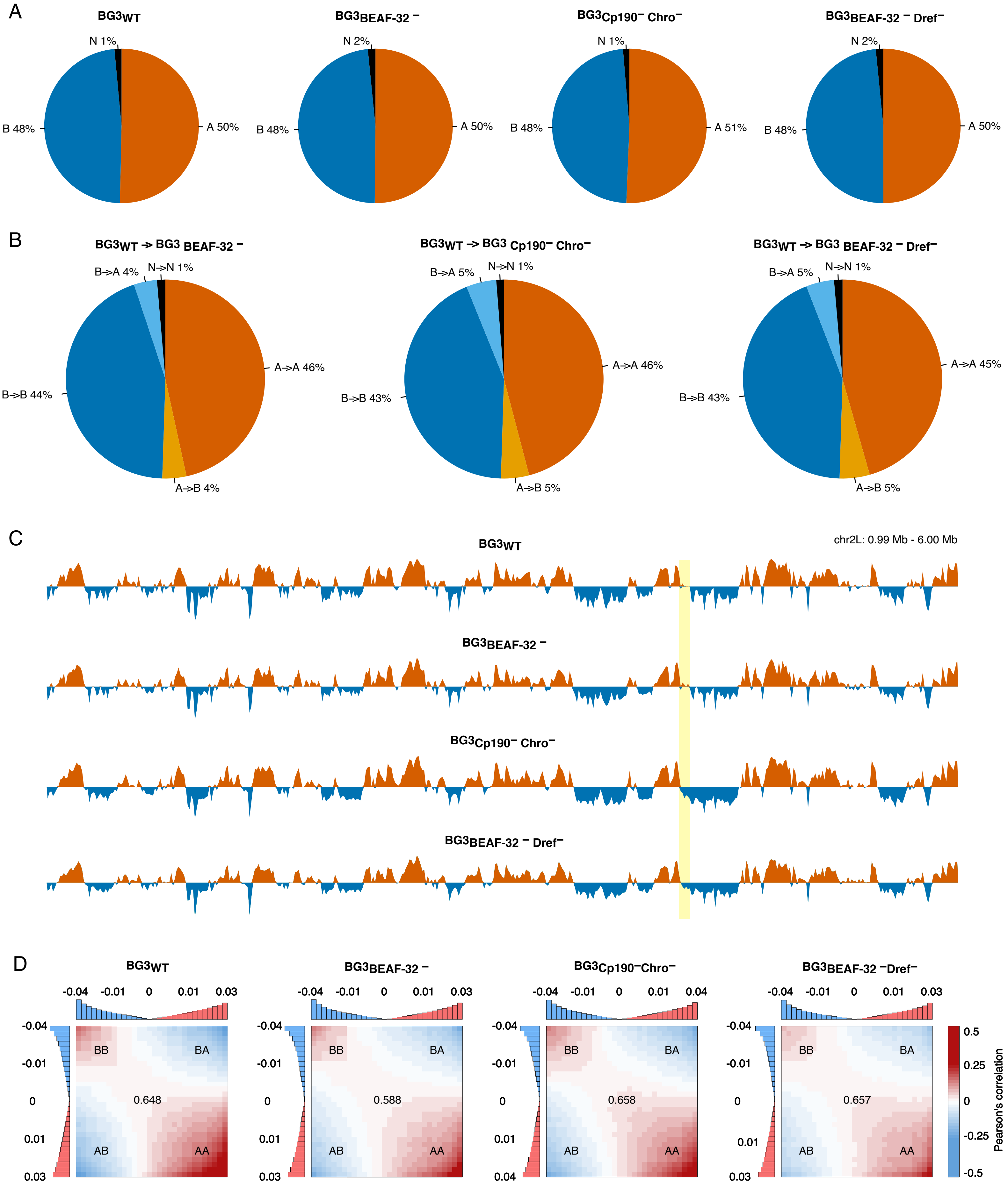

A
BG3WT
BG3BEAF-32 −
BG3Cp190− Chro−
BG3BEAF-32 − Dref−
N 1%
B 48%
A 50%
N 2%
B 48%
A 50%
N 1%
B 48%
A 51%
N 2%
B 48%
A 50%
B
BG3WT -> BG3 BEAF-32 −
BG3WT -> BG3 Cp190− Chro−
BG3WT -> BG3 BEAF-32 − Dref−
B->A 4%
N->N 1%
A->A 46%
B->B 44%
A->B 4%
B->A 5%
N->N 1%
A->A 46%
B->B 43%
A->B 5%
B->A 5%
N->N 1%
A->A 45%
B->B 43%
A->B 5%
C
BG3WT
chr2L: 0.99 Mb - 6.00 Mb
BG3BEAF-32 −
BG3Cp190− Chro−
BG3BEAF-32 − Dref−
D
BG3WT
BG3BEAF-32 −
BG3Cp190−Chro−
BG3BEAF-32 −Dref−
-0.04 -0.01 0 0.01 0.03
-0.04 -0.01 0 0.01 0.03
-0.04 -0.01 0 0.01 0.04
-0.04 -0.01 0 0.01 0.03
BB BA AB AA
0.648
0.588
0.658
0.657
0.5
0.25
0
-0.25
-0.5
Pearson's correlation

Figure S14. *A/B compartments in BG3 WT and three knockdowns.* (A) Percentage of the genome that was called as A or a B compartment in WT cells, BEAF-32 knockdown cells, Cp190 and Chro double knockdown cells and BEAF-32 and Dref double knockdown cells. The compartments were detected in 10 kb bins. Regions that could not be classified as either an A or a B compartment were labelled as N. (B) Percentage of A/B compartments that was maintained or switched in the three knockdowns. (C) Profile of eigen vectors on chromosome 2L. Red values indicated positive values (A compartment) and blue values indicate negative values (B compartment). One example of compartment switching is highlighted. (D) Averaged Pearson correlation profiles stratified by eigenvector percentile calculated as described in Methods. The top and left panels show averages over 30 percentile bins in absolute values: in blue there are partitions with negative average eigenvector scores (regions belonging to B compartments), in red there are partitions with positive average eigenvector scores (regions belonging to A compartments). Numbers at the centre of the heat maps indicate compartment strength calculated as the ratio of (AA+BB)/(AB+BA) using the mean values from corner sub-matrices of 10×10 bins.
