# Supplementary material for: The role of insulators and transcription in 3D chromatin organization of flies
Source: Genome Res. 2022 Apr;32(4):682–98. doi: 10.1101/gr.275809.121 (PMC8997359; doi:10.1101/gr.275809.121)
Supplement: Supplemental Material [file supp_gr.275809.121_Supplemental_Fig_S15.pdf]

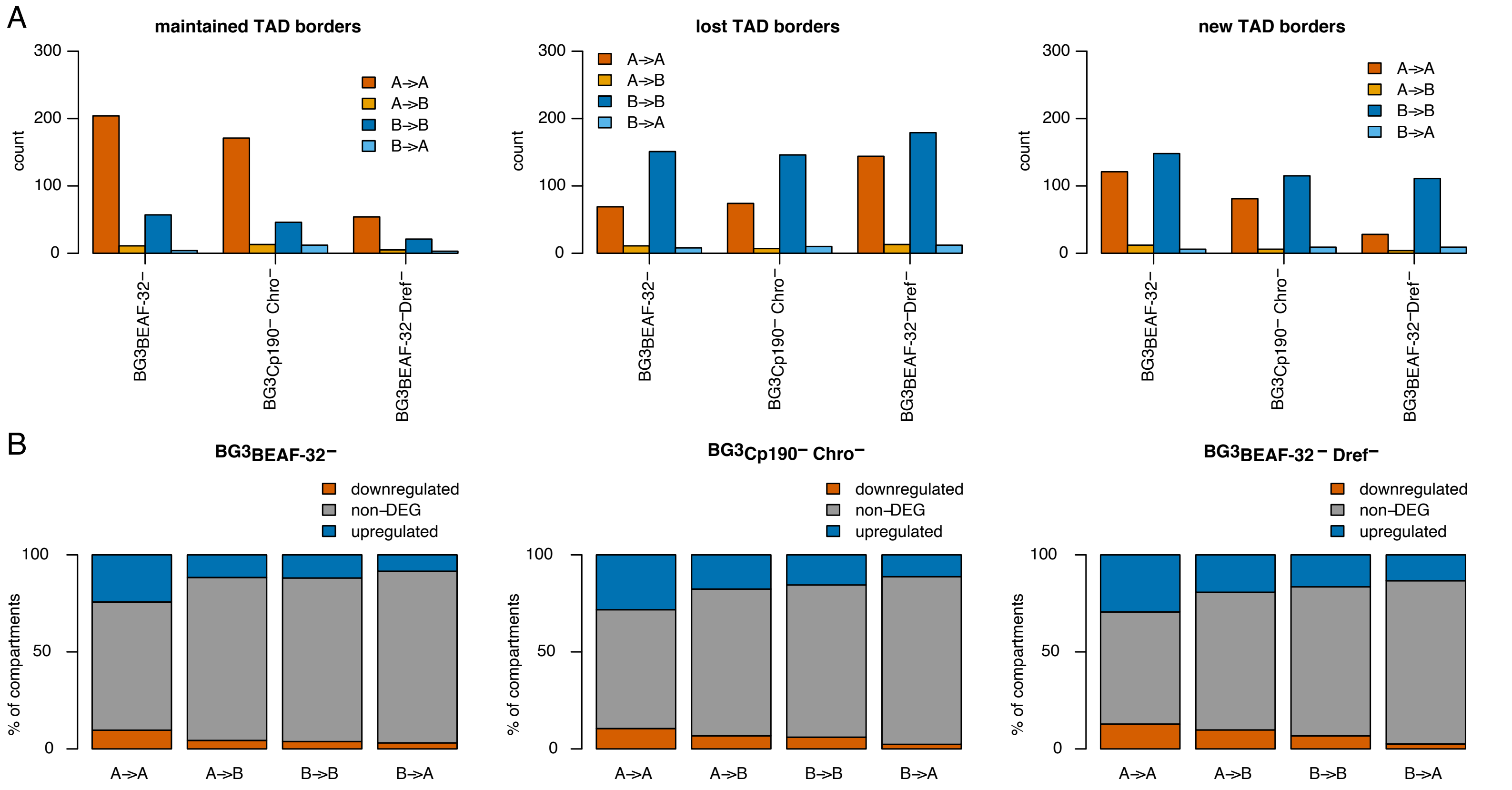
A
maintained TAD borders
lost TAD borders
new TAD borders
count
300
200
100
0
A->A
A->B
B->B
B->A
BG3BEAF-32−
BG3Cp190− Chro−
BG3BEAF-32−Dref−
B
BG3BEAF-32−
BG3Cp190− Chro−
BG3BEAF-32 − Dref−
downregulated
non–DEG
upregulated
% of compartments
100
50
0
A->A
A->B
B->B
B->A

Figure S15. *Characterisation of compartments based on TADs and differential gene expression.* (A) Distribution of maintained, lost and new TAD borders in different classes of compartments switching for the three knockdowns. (B) Percentage of compartments switching that overlap with DEG and non-DEGs. We used a $\log_2$ fold change of 1.0 when computing DEGs. Majority of compartments switch do not have any DEG.
