# Supplementary material for: The role of insulators and transcription in 3D chromatin organization of flies
Source: Genome Res. 2022 Apr;32(4):682–98. doi: 10.1101/gr.275809.121 (PMC8997359; doi:10.1101/gr.275809.121)
Supplement: Supplemental Material [file supp_gr.275809.121_Supplemental_Fig_S16.pdf]

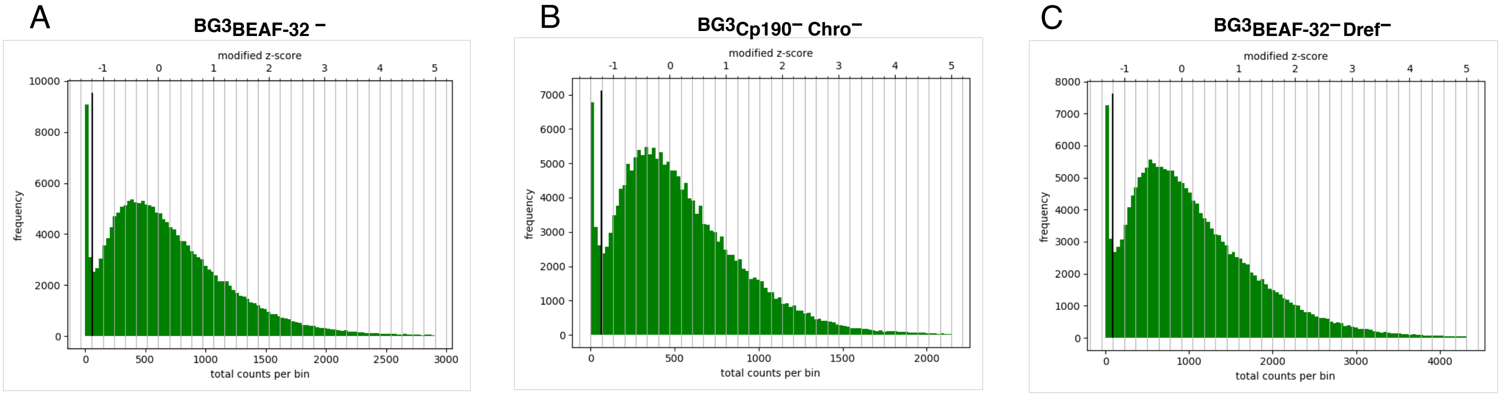
A
BG3BEAF-32 −
B
BG3Cp190− Chro−
C
BG3BEAF-32− Dref−
modified z-score
frequency
total counts per bin

Figure S16. *Diagnostic plots for correction of Hi-C plots from HiCExplorer.* Histograms of the sum of contact per bin in (A) BEAF-32 single knockdown, (B) Cp190 Chro double knockdown and (C) BEAF-32 Dref double knockdown. The vertical black line represents the lower threshold for removing bins with lower number of reads.
