# Supplementary material for: The role of insulators and transcription in 3D chromatin organization of flies
Source: Genome Res. 2022 Apr;32(4):682–98. doi: 10.1101/gr.275809.121 (PMC8997359; doi:10.1101/gr.275809.121)
Supplement: Supplemental Material [file supp_gr.275809.121_Supplemental_Table_S1.docx]

**Table S1:** *Metrics from analysis of Hi-C library sequencing*

| Sample | Library size | Source | Total reads | mappable, unique and high quality | | Pairs used | Inter-chromosomal | short range (<20kb) | long range |
| --- | --- | --- | --- | --- | --- | --- | --- | --- | --- |
| BG_WT_  replicate 1 | 100% | GSE122603 | 151,493,083 | 101,206,098 | | 42,160,814 | 7,157,538 | 12,969,585 | 22,033,691 |
| BG_WT_  replicate 2 | 100% | GSE122603 | 147,730,225 | 95,703,042 | | 42,999,219 | 7,851,274 | 13,180,675 | 21,967,270 |
| BG_BEAF-32_^-^ replicate 1 | 100% | this study | 172,597,536 | 69,859,110 | | 32,240,219 | 4,463,615 | 9,357,013 | 18,419,591 |
| BG_BEAF-32_^-^ replicate 2 | 100% | this study | 156,655,569 | 81,740,811 | | 41,458,552 | 5,690,882 | 11,839,584 | 23,928,086 |
| BG_Cp190_^-^ _Chro_^-^ replicate 1 | 100% | this study | 214,736,070 | 146,124,131 | | 65,278,295 | 8,846,046 | 18,151,528 | 38,280,721 |
| BG_Cp190_^-^ _Chro_^-^ replicate 2 | 100% | this study | 167,983,753 | 104,046,976 | | 45,830,412 | 6,142,658 | 13,111,506 | 26,576,248 |
| BG_BEAF-32_^-^ _Dref_^-^ replicate 1 | 100% | this study | 197,221,569 | 107,472,262 | | 37,738,037 | 5,085,390 | 9,902,103 | 22,750,544 |
| BG_BEAF-32_^-^ _Dref_^-^ replicate 2 | 100% | this study | 190,654,840 | 53,234,566 | | 18,645,676 | 2,462,801 | 5,210,579 | 10,972,296 |
| Kc167_WT_  replicate 1 | 100% | GSE122603 | 152,371,706 | 98,701,754 | | 49,712,803 | 3,532,342 | 16,386,181 | 29,794,280 |
| Kc167_WT_  replicate 2 | 100% | GSE122603 | 188,531,576 | 117,170,016 | | 61,211,572 | 4,522,842 | 17,549,275 | 39,139,455 |
| BG_WT_  replicate 1 - 80% | 80% | GSE122603 | 121,195,638 | | 81,045,523 | 34,528,646 | 5,857,796 | 10,713,800 | 17,957,050 |
| BG_WT_  replicate 2 - 80% | 80% | GSE122603 | 118,173,473 | | 76,656,728 | 35,462,491 | 6,480,416 | 10,908,557 | 18,073,518 |
| BG_BEAF-32_^-^ replicate 1 - 80% | 80% | this study | 138,073,566 | | 55,898,357 | 26,805,854 | 3,703,534 | 7,833,118 | 15,269,202 |
| BG_BEAF-32_^-^ replicate 2 - 80% | 80% | this study | 125,326,239 | | 65,443,211 | 33,860,610 | 4,647,796 | 9,764,035 | 19,448,779 |
| BG_Cp190_^-^ _Chro_^-^ replicate 1 - 80% | 80% | this study | 171,799,624 | | 117,064,857 | 53,376,413 | 7,234,167 | 14,861,363 | 31,280,883 |
| BG_Cp190_^-^ _Chro_^-^ replicate 2 - 80% | 80% | this study | 134,385,744 | | 83,338,488 | 37,245,423 | 4,993,379 | 10,680,858 | 21,571,186 |
| BG_BEAF-32_^-^ _Dref_^-^ replicate 1 - 80% | 80% | this study | 157,771,608 | | 86,029,067 | 31,775,241 | 4,278,328 | 8,338,702 | 19,158,211 |
| BG_BEAF-32_^-^ _Dref_^-^ replicate 2 - 80% | 80% | this study | 152,530,267 | | 42,612,149 | 15,651,046 | 2,066,595 | 4,379,973 | 9,204,478 |
| Kc167_WT_  replicate 1 - 80% | 80% | GSE122603 | 121,887,879 | | 78,989,128 | 40,649,289 | 2,888,640 | 13,471,045 | 24,289,604 |
| Kc167_WT_  replicate 2 - 80% | 80% | GSE122603 | 150,825,628 | | 93,798,562 | 50,505,474 | 3,752,258 | 14,525,871 | 32,227,345 |
