# Supplementary material for: The role of insulators and transcription in 3D chromatin organization of flies
Source: Genome Res. 2022 Apr;32(4):682–98. doi: 10.1101/gr.275809.121 (PMC8997359; doi:10.1101/gr.275809.121)
Supplement: Supplemental Material [file supp_gr.275809.121_Supplemental_Table_S2.docx]

**Table S2:** *Parameters for the correction of Hi-C matrices and the number of TADs*

| Sample | Library Size | Threshold DpnII | Threshold 10 Kb | Threhsold 100 Kb | Number of TAD borders | Number of strong TAD border |
| --- | --- | --- | --- | --- | --- | --- |
| BG_WT_  replicate 1 | 100% | [-1.4, 5] | [-1.4, 5] | [-2.4, 5] |  |  |
| BG_WT_  replicate 2 | 100% | [-1.4, 5] | [-1.4, 5] | [-2.4, 5] |  |  |
| BG_WT_  merged | 100% | [-1.4, 5] | [-1.4, 5] | [-2.4, 5] | 2260 | 989 |
| BG_BEAF-32_^-^ replicate 1 | 100% | [-1.0, 5] | [-0.8, 5] | [-1.9, 5] |  |  |
| BG_BEAF-32_^-^ replicate 2 | 100% | [-1.2, 5] | [-1.4, 5] | [-2.2, 5] |  |  |
| BG_BEAF-32_^-^ merged | 100% | [-1.2, 5] | [-1.4, 5] | [-2.2, 5] | 2557 | 1136 |
| BG_Cp190_^-^ _Chro_^-^ replicate 1 | 100% | [-1.2, 5] | [-1.4, 5] | [-2.4, 5] |  |  |
| BG_Cp190_^-^ _Chro_^-^ replicate 2 | 100% | [-1.2, 5] | [-1.4, 5] | [-2.4, 5] |  |  |
| BG_Cp190_^-^ _Chro_^-^ merged | 100% | [-1.2, 5] | [-1.4, 5] | [-2.4, 5] | 2163 | 869 |
| BG_BEAF-32_^-^ _Dref_^-^ replicate 1 | 100% | [-1.2, 5] | [-1.4, 5] | [-2.4, 5] |  |  |
| BG_BEAF-32_^-^ _Dref_^-^ replicate 2 | 100% | [-1.2, 5] | [-1.4, 5] | [-2.4, 5] |  |  |
| BG_BEAF-32_^-^ _Dref_^-^ merged | 100% | [-1.2, 5] | [-1.4, 5] | [-2.4, 5] | 1417 | 441 |
| Kc167_WT_  replicate 1 | 100% | [-1.4, 5] | [-1.4, 5] | [-2.6, 5] |  |  |
| Kc167_WT_  replicate 2 | 100% | [-1.4, 5] | [-2.0, 5] | [-3.0, 5] |  |  |
| Kc167_WT_  merged | 100% | [-1.4, 5] | [-1.6, 5] | [-3.0, 5] | 2512 | 1306 |
| BG_WT_  replicate 1 - 80% | 80 % |  |  |  |  |  |
| BG_WT_  replicate 2 - 80% | 80 % |  |  |  |  |  |
| BG_WT_  merged - 80% | 80 % | [-1.4, 5] |  |  | 2179 | 902 |
| BG_BEAF-32_^-^ replicate 1 - 80% | 80 % |  |  |  |  |  |
| BG_BEAF-32_^-^ replicate 2 - 80% | 80 % |  |  |  |  |  |
| BG_BEAF-32_^-^ merged - 80% | 80 % | [-1.2, 5] |  |  | 2493 | 1074 |
| BG_Cp190_^-^ _Chro_^-^ replicate 1 - 80% | 80 % |  |  |  |  |  |
| BG_Cp190_^-^ _Chro_^-^ replicate 2 - 80% | 80 % |  |  |  |  |  |
| BG_Cp190_^-^ _Chro_^-^ merged - 80% | 80 % | [-1.2, 5] |  |  | 2051 | 776 |
| BG_BEAF-32_^-^ _Dref_^-^ replicate 1 - 80% | 80 % |  |  |  |  |  |
| BG_BEAF-32_^-^ _Dref_^-^ replicate 2 - 80% | 80 % |  |  |  |  |  |
| BG_BEAF-32_^-^ _Dref_^-^ merged - 80% | 80 % | [-1.2, 5] |  |  | 1346 | 416 |
| Kc167_WT_  replicate 1 - 80% | 80 % |  |  |  |  |  |
| Kc167_WT_  replicate 2 - 80% | 80 % |  |  |  |  |  |
| Kc167_WT_  merged - 80% | 80 % | [-1.4, 5] |  |  | 2479 | 1229 |
