# Supplementary material for: The role of insulators and transcription in 3D chromatin organization of flies
Source: Genome Res. 2022 Apr;32(4):682–98. doi: 10.1101/gr.275809.121 (PMC8997359; doi:10.1101/gr.275809.121)
Supplement: Supplemental Material [file supp_gr.275809.121_Supplemental_Table_S3.docx]

**Table S3:** *Metrics from analysis of RNA-seq library sequencing*

| Sample | Total reads | After trimming | Aligned pairs | Concordant alignment rate | Multiple alignments | DEG(log_2_FC=2.0) |
| --- | --- | --- | --- | --- | --- | --- |
| BG_WT_  replicate 1 | 24,486,765 | 23,334,081 | 19,022,098 | 80.2% | 15.9% |  |
| BG_WT_  replicate 2 | 24,953,334 | 23,902,047 | 19,891,746 | 82.0% | 14.9% |  |
| BG_WT_  replicate 3 | 25,765,693 | 24,761,665 | 20,804,226 | 82.4% | 15.3% |  |
| BG_BEAF-32_^-^ replicate 1 | 22,783,325 | 21,691,464 | 17,512,866 | 78.8% | 20.5% | 596 |
| BG_BEAF-32_^-^ replicate 2 | 26,110,498 | 25,485,051 | 21,761,711 | 83.6% | 20.1% |  |
| BG_BEAF-32_^-^ replicate 3 | 22,390,841 | 21,402,262 | 17,408,320 | 78.5% | 29.1% |  |
| BG_Cp190_^-^ _Chro_^-^ replicate 1 | 29,595,777 | 27,953,418 | 22,271,222 | 77.6% | 22.5% | 687 |
| BG_Cp190_^-^ _Chro_^-^ replicate 2 | 25,622,361 | 24,395,295 | 19,674,867 | 78.6% | 21.8% |  |
| BG_Cp190_^-^ _Chro_^-^ replicate 3 | 33,314,606 | 32,443,121 | 27,496,068 | 82.9% | 21.6% |  |
| BG_BEAF-32_^-^ _Dref_^-^ replicate 1 | 30,470,304 | 29,551,901 | 25,119,383 | 83.1% | 23.8% | 810 |
| 0BG_BEAF-32_^-^ _Dref_^-^ replicate 2 | 21,519,093 | 20,564,923 | 17,119,372 | 81.2% | 24.2% |  |
| BG_BEAF-32_^-^ _Dref_^-^ replicate 3 | 25,156,762 | 23,927,406 | 19,266,708 | 77.9% | 25.0% |  |
