# Supplementary material for: The role of insulators and transcription in 3D chromatin organization of flies
Source: Genome Res. 2022 Apr;32(4):682–98. doi: 10.1101/gr.275809.121 (PMC8997359; doi:10.1101/gr.275809.121)
Supplement: Supplemental Material [file supp_gr.275809.121_Supplemental_Table_S4.docx]

**Table S4:** *Classification of genes located at maintained TAD borders*. We considered all maintained TAD borders that are common between BEAF-32 single mutant and Cp190 and Chro double mutant and that are also present in Kc167 cells (159 borders). We selected all genes within 5 Kb (257) and then identified their expression levels in 85 tissues/developmental times or cell lines (252 genes). Genes that were expressed in 40th percentile of expression in all 85 samples were classified as house keeping genes.

| **gene** | **expressed** | **housekeeping** |
| --- | --- | --- |
| galectin | 85 | Yes |
| CG11374 | 85 | Yes |
| MED15 | 85 | Yes |
| cbt | 85 | Yes |
| CG15358 | 85 | Yes |
| CG42296 | 85 | Yes |
| lectin-22C | 85 | Yes |
| Su(dx) | 85 | Yes |
| CG10874 | 85 | Yes |
| CG34172 | 85 | Yes |
| cype | 85 | Yes |
| CG14022 | 85 | Yes |
| TotM | 85 | Yes |
| fusl | 85 | Yes |
| frj | 85 | Yes |
| CG16947 | 85 | Yes |
| CG33129 | 85 | Yes |
| rho-6 | 85 | Yes |
| Gr33a | 85 | Yes |
| pkaap | 85 | Yes |
| CG17329 | 85 | Yes |
| CG13258 | 85 | Yes |
| RpL30 | 85 | Yes |
| robl37BC | 85 | Yes |
| brat | 85 | Yes |
| CG10188 | 85 | Yes |
| CG10186 | 85 | Yes |
| CG10481 | 85 | Yes |
| CG31619 | 85 | Yes |
| CG30497 | 85 | Yes |
| Camta | 85 | Yes |
| Jra | 85 | Yes |
| RpLP0-like | 85 | Yes |
| 14-3-3zeta | 85 | Yes |
| E(Pc) | 85 | Yes |
| Drep-1 | 85 | Yes |
| RpS11 | 85 | Yes |
| CG8331 | 85 | Yes |
| mRpS16 | 85 | Yes |
| cg | 85 | Yes |
| dup | 85 | Yes |
| SRPK | 85 | Yes |
| CG8249 | 85 | Yes |
| tun | 85 | Yes |
| GEFmeso | 85 | Yes |
| CG42855 | 85 | Yes |
| Sik3 | 85 | Yes |
| CG15087 | 85 | Yes |
| Nnf1a | 85 | Yes |
| bl | 85 | Yes |
| otp | 85 | Yes |
| Sara | 85 | Yes |
| CG13500 | 85 | Yes |
| CG13501 | 85 | Yes |
| Snp | 85 | Yes |
| RpS16 | 85 | Yes |
| Vps20 | 85 | Yes |
| CG4329 | 85 | Yes |
| CG7049 | 85 | Yes |
| Vdup1 | 85 | Yes |
| CG13875 | 85 | Yes |
| CG32295 | 85 | Yes |
| msn | 85 | Yes |
| CG9977 | 85 | Yes |
| CG2162 | 85 | Yes |
| CG17746 | 85 | Yes |
| CG12078 | 85 | Yes |
| CG5568 | 85 | Yes |
| CG18586 | 85 | Yes |
| CG18769 | 85 | Yes |
| bin | 85 | Yes |
| CG8281 | 85 | Yes |
| CG8111 | 85 | Yes |
| CG32368 | 85 | Yes |
| HP4 | 85 | Yes |
| CG8209 | 85 | Yes |
| CG8038 | 85 | Yes |
| nmo | 85 | Yes |
| eIF-4E | 85 | Yes |
| Cpr67B | 85 | Yes |
| CG32100 | 85 | Yes |
| nan | 85 | Yes |
| Baldspot | 85 | Yes |
| Dab | 85 | Yes |
| nudC | 85 | Yes |
| CG13024 | 85 | Yes |
| CG7589 | 85 | Yes |
| CG7580 | 85 | Yes |
| CG34250 | 85 | Yes |
| CG13733 | 85 | Yes |
| qjt | 85 | Yes |
| Nedd4 | 85 | Yes |
| CG34251 | 85 | Yes |
| MESR6 | 85 | Yes |
| CG11577 | 85 | Yes |
| CG10424 | 85 | Yes |
| HLH106 | 85 | Yes |
| CG7365 | 85 | Yes |
| gig | 85 | Yes |
| obst-J | 85 | Yes |
| CG7335 | 85 | Yes |
| CG40249 | 85 | Yes |
| CG42617 | 85 | Yes |
| CG14657 | 85 | Yes |
| CG12007 | 85 | Yes |
| CG2993 | 85 | Yes |
| CG31472 | 85 | Yes |
| CG31473 | 85 | Yes |
| CG34384 | 85 | Yes |
| CG10092 | 85 | Yes |
| mRpS9 | 85 | Yes |
| RnpS1 | 85 | Yes |
| CG9386 | 85 | Yes |
| CG8199 | 85 | Yes |
| CG8358 | 85 | Yes |
| CG16817 | 85 | Yes |
| nmdyn-D7 | 85 | Yes |
| CG9444 | 85 | Yes |
| trbd | 85 | Yes |
| CG8534 | 85 | Yes |
| eloF | 85 | Yes |
| CG3940 | 85 | Yes |
| CG14710 | 85 | Yes |
| CG6808 | 85 | Yes |
| CG14711 | 85 | Yes |
| CG31211 | 85 | Yes |
| Vha55 | 85 | Yes |
| Snx3 | 85 | Yes |
| kibra | 85 | Yes |
| CG42788 | 85 | Yes |
| mor | 85 | Yes |
| Hel89B | 85 | Yes |
| CG31287 | 85 | Yes |
| mtSSB | 85 | Yes |
| bor | 85 | Yes |
| asun | 85 | Yes |
| Pxt | 85 | Yes |
| Atpalpha | 85 | Yes |
| tin | 85 | Yes |
| rdhB | 85 | Yes |
| unk | 85 | Yes |
| VhaAC39-2 | 85 | Yes |
| CG13829 | 85 | Yes |
| CG6763 | 85 | Yes |
| CG34355 | 85 | Yes |
| jigr1 | 85 | Yes |
| CG6142 | 85 | Yes |
| scrib | 85 | Yes |
| ms(3)K81 | 85 | Yes |
| ro | 85 | Yes |
| CG5500 | 85 | Yes |
| T48 | 85 | Yes |
| Ets97D | 85 | Yes |
| CG11873 | 85 | Yes |
| CG31019 | 85 | Yes |
| CG31021 | 85 | Yes |
| PH4alphaNE3 | 85 | Yes |
| CG31016 | 85 | Yes |
| CG15561 | 85 | Yes |
| CG1746 | 85 | Yes |
| CG12054 | 85 | Yes |
| RpS3A | 85 | Yes |
| pan | 85 | Yes |
| fd102C | 85 | Yes |
| CG3556 | 85 | Yes |
| ras | 85 | Yes |
| Rph | 85 | Yes |
| Atg8a | 85 | Yes |
| CG9919 | 85 | Yes |
| CG9921 | 85 | Yes |
| Dsp1 | 85 | Yes |
| EF2 | 85 | Yes |
| CG1832 | 85 | Yes |
| CG1240 | 85 | Yes |
| Sar1 | 85 | Yes |
| CG1826 | 85 | Yes |
| RapGAP1 | 84 | No |
| CG18616 | 84 | No |
| Cdc16 | 82 | No |
| CG1401 | 52 | No |
| CG9236 | 41 | No |
| Obp28a | NA | No |
| CG45093 | NA | No |
| CG45086 | NA | No |
| Galphaq | NA | No |
| CG44433 | NA | No |
