# Supplementary material for: The role of insulators and transcription in 3D chromatin organization of flies
Source: Genome Res. 2022 Apr;32(4):682–98. doi: 10.1101/gr.275809.121 (PMC8997359; doi:10.1101/gr.275809.121)
Supplement: Supplemental Material [file supp_gr.275809.121_Supplemental_Table_S5.docx]

**Table S5:** *Primer sequences used for RNAi and RT-qPCR.*

| **RNAi primer sequences** | |
| --- | --- |
| BEAF-32_F(RNAi) | TAATACGACTCACTATAGGGGGAGGAGTACGAGCAGAACG |
| BEAF-32_R(RNAi) | TAATACGACTCACTATAGGGACGCTGATTTGCCCATTTAC |
| Chro_F(RNAi) | TAATACGACTCACTATAGGGCTTTGTTATTCGCACAGGCA |
| Chro_R(RNAi) | TAATACGACTCACTATAGGGCAGGAGGAATTGGCAAACAT |
| Cp190_F(RNAi) | TAATACGACTCACTATAGGGCGGCATGGACATCATCATAA |
| Cp190_R(RNAi) | TAATACGACTCACTATAGGGACAGTTGGACAGCCCAGTTC |
| Dref_F(RNAi) | TAATACGACTCACTATAGGGCGAGATACCAAATCCTCCGA |
| Dref_R(RNAi) | TAATACGACTCACTATAGGGTCGCCAGTGCAGACTAATTG |
| **RT-qPCR primer sequences** | |
| BEAF-32_F | AGGATCCACTGTGCTATAGTCC |
| BEAF-32_R | GCTGGTGAAGTCGAATGGGT |
| Chro_F | AGTTTAAAGCTATCGACAGG |
| Chro_R | CAGAGATGATTTGGTTCCG |
| Cp190_F | CACCGACTACTTCAATGTAC |
| Cp190_R | TTTTAAGCTCAAACTCCAGG |
| Dref_F | CCCAAGATGAAAAGCGTATA |
| Dref_R | CCTTGTGACACTTAATGCAGA |
| RpL32_F | AAGCGGCGACGCACTCTGTT |
| RpL32_R | GCCCAGCATACAGGCCCAAG |
