# Supplementary material for: The role of insulators and transcription in 3D chromatin organization of flies
Source: Genome Res. 2022 Apr;32(4):682–98. doi: 10.1101/gr.275809.121 (PMC8997359; doi:10.1101/gr.275809.121)
Supplement: Supplemental Material [file supp_gr.275809.121_Supplemental_Table_S6.docx]

**Table S6:** *Datasets for architectural proteins used in this work*

| **Architectural proteins** | | | **dm3 or dm6** | **LiftOver to dm6** |
| --- | --- | --- | --- | --- |
| BEAF-32 | 921 | GSE20811 | dm3 | yes |
| CTCF | 3673 | GSE32783 | dm3 | yes |
| CP190 | 924 | GSE20814 | dm3 | yes |
| Criz/Chro | 275 | [GSE20761](https://www.ncbi.nlm.nih.gov/geo/query/acc.cgi?acc=GSE20761) | dm3 | yes |
| Trl | 2651 | [GSE23466](https://www.ncbi.nlm.nih.gov/geo/query/acc.cgi?acc=GSE23466) | dm3 | yes |
| JIL-1 | 3035 | GSE27754 | dm3 | yes |
| mod(mdg4) | 324 | GSE20802 | dm3 | yes |
| su(Hw) | 951 | GSE20833 | dm3 | yes |
| Zw5 | 3064 | GSE25373 | dm3 | yes |
| fs(1)h | Pherson et al (2019) | GSE118484 | dm3 | yes |
| NippedB | Pherson et al (2019) | GSE118484 | dm3 | yes |
| Rad21 | Pherson et al (2019) | GSE118484 | dm3 | yes |
| SA | Pherson et al (2019) | GSE118484 | dm3 | yes |
| SMC1 | Pherson et al (2019) | GSE118484 | dm3 | yes |
