# Supplementary material for: The role of insulators and transcription in 3D chromatin organization of flies
Source: Genome Res. 2022 Apr;32(4):682–98. doi: 10.1101/gr.275809.121 (PMC8997359; doi:10.1101/gr.275809.121)
Supplement: Supplemental Material [file supp_gr.275809.121_Supplemental_Table_S7.docx]

**Table S7:** *Datasets for transcription and replication used in this work*

| **Transcription and replication** | | | **dm3 or dm6** | **LiftOver to dm6** |
| --- | --- | --- | --- | --- |
| Orc2 | 2754 | GSE20888 | dm3 | yes |
| Topo-II | 5058 | GSE45069 | dm3 | yes |
| Pof | 3052 | GSE27808 | dm3 | yes |
| Pol-II | 950 | [GSE20832](https://www.ncbi.nlm.nih.gov/geo/query/acc.cgi?acc=GSE20832) | dm3 | yes |
| 3'NT-seq | Pherson et al (2017) | GSE100545 | dm3 | yes |
| MED1 | Pherson et al (2019) | GSE118484 | dm3 | yes |
| MED30 | Pherson et al (2019) | GSE118484 | dm3 | yes |
