# Supplementary material for: The role of insulators and transcription in 3D chromatin organization of flies
Source: Genome Res. 2022 Apr;32(4):682–98. doi: 10.1101/gr.275809.121 (PMC8997359; doi:10.1101/gr.275809.121)
Supplement: Supplemental Material [file supp_gr.275809.121_Supplemental_Table_S8.docx]

**Table S8:** *Datasets for DNA accessibility used in this work*

| **DNA accessibility** | | | **dm3 or dm6** | **LiftOver to dm6** |
| --- | --- | --- | --- | --- |
| DNase-I | Kharchenko et al (2011) | - | dm3 | yes |
| H1 | 3299 | GSE32767 | dm3 | yes |
| H2Av | 6073 | GSE45110 | dm3 | yes |
| H3 | 3302 | GSE32769 | dm3 | yes |
| H4 | 3303 | GSE32770 | dm3 | yes |
