# Supplementary material for: The role of insulators and transcription in 3D chromatin organization of flies
Source: Genome Res. 2022 Apr;32(4):682–98. doi: 10.1101/gr.275809.121 (PMC8997359; doi:10.1101/gr.275809.121)
Supplement: Supplemental Material [file supp_gr.275809.121_Supplemental_Table_S9.docx]

**Table S9:** *Datasets for histone modifications used in this work*

| **Histone modificcations** | | | **dm3 or dm6** | **LiftOver to dm6** |
| --- | --- | --- | --- | --- |
| H2Bubi | 288 | GSE20771 | dm3 | yes |
| H3K18ac | 291 | GSE20774 | dm3 | yes |
| H3K23ac | 293 | GSE20776 | dm3 | yes |
| H3K27ac | 295 | GSE20778 | dm3 | yes |
| H3K27me1 | 3941 | GSE51965 | dm3 | yes |
| H3K27me2 | 2999 | GSE27789 | dm3 | yes |
| H3K27me3 | 297 | GSE20780 | dm3 | yes |
| H3K36me1 | 299 | GSE20782 | dm3 | yes |
| H3K36me3 | 301 | GSE20783 | dm3 | yes |
| H3K4me1 | 2653 | GSE23468 | dm3 | yes |
| H3K4me2 | 2654 | GSE23469 | dm3 | yes |
| H3K4me3 | 967 | GSE20839 | dm3 | yes |
| H3K79me1 | 3005 | GSE32736 | dm3 | yes |
| H3K79me2 | 306 | GSE20788 | dm3 | yes |
| H3K79me3 | 4934 | GSE45062 | dm3 | yes |
| H3K9me2 | 310 | GSE20791 | dm3 | yes |
| H3K9me3 | 312 | GSE20793 | dm3 | yes |
| H4K16ac | 316 | GSE20795 | dm3 | yes |
| H4K20me1 | 3286 | GSE32755 | dm3 | yes |
| H4K8ac | 5060 | GSE45070 | dm3 | yes |
