# Supplementary material for: The role of insulators and transcription in 3D chromatin organization of flies
Source: Genome Res. 2022 Apr;32(4):682–98. doi: 10.1101/gr.275809.121 (PMC8997359; doi:10.1101/gr.275809.121)
Supplement: Supplemental Material [file supp_gr.275809.121_Supplemental_Table_S10.docx]

**Table S10:** *Datasets for nucleosome remodelling factors used in this work*

| **Nucleosome remodelling factors** | | | **dm3 or dm6** | **LiftOver to dm6** |
| --- | --- | --- | --- | --- |
| ash1 | 3279 | GSE32748 | dm3 | yes |
| Kdm2 | 5145 | GSE45092 | dm3 | yes |
| MRG15 | 3045 | GSE25365 | dm3 | yes |
| NURF301 | 5063 | GSE45072 | dm3 | yes |
| PR-Set7 | 5065 | GSE45074 | dm3 | yes |
| RPD3 | 4188 | GSE44523 | dm3 | yes |
| wds | 5148 | GSE45094 | dm3 | yes |
| Iswi | 3030 | GSE27750 | dm3 | yes |
| mof | 3041 | GSE27803 | dm3 | yes |
