# Supplementary material for: The role of insulators and transcription in 3D chromatin organization of flies
Source: Genome Res. 2022 Apr;32(4):682–98. doi: 10.1101/gr.275809.121 (PMC8997359; doi:10.1101/gr.275809.121)
Supplement: Supplemental Material [file supp_gr.275809.121_Supplemental_Table_S11.docx]

**Table S11:** *Datasets for polycomb and heterochromatin used in this work*

| **Polycomb and heterochromatin** | | | **dm3 or dm6** | **LiftOver to dm6** |
| --- | --- | --- | --- | --- |
| Pc | 325 | GSE20803 | dm3 | yes |
| dRING | 927 | GSE20817 | dm3 | yes |
| Sfmbt | 2986 | GSE27728 | dm3 | yes |
| E(z) | 2650 | GSE23465 | dm3 | yes |
| Pcl | 948 | GSE20830 | dm3 | yes |
| Psc | 3055 | GSE25370 | dm3 | yes |
| HP1a | 4126 | GSE44515 | dm3 | yes |
| HP1b | 3016 | GSE44462 | dm3 | yes |
| HP1c | 942 | GSE20824 | dm3 | yes |
| HP2 | 3026 | GSE27747 | dm3 | yes |
| HP4 | 4185 | GSE44521 | dm3 | yes |
| Su(var)3-7 | 2671 | GSE23486 | dm3 | yes |
| Su(var)3-9 | 952 | GSE20834 | dm3 | yes |
